# Advanced deep learning framework for soil texture classification

**DOI:** 10.1038/s41598-025-17384-5

**Published:** 2025-10-02

**Authors:** N. Latha Reddy, M.P. Gopinath

**Affiliations:** School of Computer Science and Engineering, Vellore Institute of Technology, Vellore, TamilNadu India

**Keywords:** Soil texture classification, Farthing histogram of oriented gradients (F-HOG), Optimization algorithm, EWJFO, Deep learning-based detection, ATFEM, VGG-RTPNet, ResNet-DANet, Swin-FANet, Computational biology and bioinformatics, Solid Earth sciences, Engineering

## Abstract

In soil texture classification, accuracy with interpretability is the key to sustainable agriculture and environmental management. The presented ATFEM (Advanced Triptych Feature Engineering and Modeling framework) framework synergizes handcrafted texture features with learned deep representations through a three-stream architecture: VGG-RTPNet (Residual Texture-Preserving Network based on Visual Geometry Group-16) for texture, ResNet-DANet (Residual Network integrated with Dual Attention Network) for semantics, and Swin-FANet (Shifted Window-based Frequency-Aware Network based on Transformer) for spectral spatial correlation. Subsequently, these branches help in extracting fine-grained structural, dual-attention-enhanced semantic, and spectral-spatial correlation-wise features of soil-image data. To further eliminate redundancy from the feature sets and arrive at the best representation, a Feature Fusion and Selection strategy employing an enhanced hybrid metaheuristic method termed EWJFO (Enhanced Wombat-Jellyfish Feature Optimization) is proposed. It synthesizes the adaptive exploration behavior of Wombat Optimization Algorithm (WOA) with the swift control convergence tempo of the Jellyfish Search Optimizer (JSO) to select the best feature subset. In addition, a new handcrafted descriptor for soil texture image analysis referred as Farthing Ornament of Histogram of Oriented Gradients (F-HOG) has been introduced with adapative. Conventional HOG is burdened with having high-dimensional redundancy and hence suffers from noise sensitivity, F-HOG combines the effect of a Butterworth frequency filter to remove the unwanted high-frequency artifacts and then goes on to perform the statistical selection of the most frequent gradient bins, thus reducing dimensions and retaining quite a bit of the discriminative structural information. The experiments were conducted on a self-built soil texture image dataset consisting of 4,000 labeled images distributed among five texture classes. ATFEM achieved an accuracy of 98.10%, an F1 score of 89.60%, Cohen’s kappa rating of 94.80%, and an AUC of 98.10%, outperforming cutting-edge methods such as CatBoost-DNN, GBDT-CNN, and SVC-RF. This work offers an upscalable, explainable, and expressively accurate solution for soil texture mapping in precision agriculture and environmental monitoring.

**Supplementary Information:**

The online version contains supplementary material available at 10.1038/s41598-025-17384-5.

## Introduction

The surface texture of the soil has a substantial effect on soil deterioration, conveyance by water, productivity of soil, and soil quality control. Large samples of soil must be collected for analysis in order to evaluate the variety of soil texture. Although the estimated amount of soil materials can be developed in a number of ways, field experiments are presently employed to produce implicit assessment that^1^ utilize remotely located sensors. Infrared and shortwave infrared (SWIR) spectroscopy are commonly used to estimate soil properties through chemometric techniques. These approaches have been employed in previous centuries because they are thought to be an inexpensive way to estimate soil characteristics primarily on their absorption range, which is 400–2500 nm. In order to comprehend and grasp the connections between further data sets, artificial neural networks are utilized for data training. A neural network, or Neural networks are models for computing that use basic nodes for processing information. It facilitates the development of computational techniques which mimic the activities of the human brain. To achieve the desired result, the weights are modified or trained for specific values. In soil research, neural networks^2^ which are made up of input, hidden, and output units^3^ are frequently used to predict the characteristics of soil. The categorizing procedure aids in the collection of more data regarding physical and chemical characteristics that require further examination to determine^4^ the value of their assets. The organic materials in the soil can be identified by their color and texture. Because of its great efficiency and dependability, digital soil mapping (DSM) became developed as an alternative to costly and time-consuming field surveys for forecasting soil attributes^5^. Because of their high return rate, quality, and availability at many scales, as well as their timeliness, affordability, and practicality, geographical pictures are appealing and crucial data in DSM^6^. In order to get textural knowledge obtained through spectroscopic regarding the reflecting properties of bare soil pixels, the majority of researchers employ remote sensing^7^. These studies are limited to arid and semi-arid locations with sparse vegetation^8^, cropland under fallow or foundation conditions^9^, or places with bare soils detected by time series multispectral imagery^10^, and they mostly depend on the availability of bare soil.

In extensively populated regions (such as forest systems and tropical hillslope environments), some works have begun to determine the texture of soil employing remote-sensed vegetation indices alongside additional factors (such as geography and stratum) because satellite images (such as Landsat and MODIS) are also capable of describing trees the characteristics as replacements for soil attributes inference^11,12^. These studies show that multitemporal optical images collecting substantial agricultural development information are beneficial for distinguishing soil texture classes^13^ and that the addition of satellite variables indicating vegetation features could increase the accuracy of soil texture prediction^14^. Since intensive farming is common in the continental the monsoon temperature zone, it is challenging to detect signs of exposed soils. To map the spatial variance of soil texture, more detailed vegetative knowledge is required^31,32^.

Numerous statistical approaches, such as multiple logistic regression^15^, geographical statistical techniques^16^, and machine learning, or ML, modelling^17^, have been employed to forecast soil attributes based on the DSM framework^18^. Because machine learning (ML) algorithms, like random forest (RF)^19^, classification and regression trees^20^, and support vector machines (SVM)^23^, can learn unpredictable relationships iteratively from data without possibly losing related information, they usually do better in predictions. SVM is well-known for its strong adaptation capabilities in machine learning algorithms. It has a solid theoretical foundation and looks for the optimal balance between model complexity and fitting accuracy using the structure risk minimization concept.

Classification of soil texture is one of the most fundamental tools in agricultural practices, land use, and management and sustainable practices because it defines issues to do with water infiltration and holding capacity and nutrient supply, choice of crops, and health of the soil. Conventional methods are used such as the hydrometer and pipette and are precise and sensitive but time-consuming, expensive and not suitable for large samples or high sample turn around^31^. Although imaging-based methods are quicker, these traditional approaches are based under controlled environment where the light is well defined and the samples are preprepared and free from any field related problems like irregular lighting, shadows, moisture and even interference by vegetation cover. Moreover, the technique of large-scale soil texture mapping is hampered by the availability of spatial data of limited geographical detail and the efficiency of synthesizing disparate environmental indices including topography, climate, and remote sensing data. Deep learning^33^ and more generally machine learning are recent approaches for which existing models often present problems of adaptation to heterogeneous data bases, accuracy for different types of soils and generalization over different world regions. Furthermore, the split approaches, which consist of fine-grained classification, such as using convolutional neural networks, or large-scale regionalization, such as Random Forest models, are not a synthesis of the two effective approaches. An integrated and scalable application closely related to these gaps is to develop methods that employ superior modern computational techniques such as image processing, transfer learning and multispectral center remote sensing data to address the above limitations to support accurate efficient and scalable segmentation and classification of soil texture in different contexts. The contribution of the work is as follows,

Major Contributions and Novelties of the Study.

- Introduction of Farthing Histogram-Oriented Gradients (F-HOG): A novel feature descriptor applies a Butterworth frequency-domain filter followed by frequency-based bin pruning, thereby achieving an extreme level of reduction in dimensionality but still maintaining the bulk of gradient information.
- ϕ-Pixels Statistical Filtering Mechanism: A frequency-based pixel selection method that assesses and retains the top pixels considered to be the most statistically significant according to a histogram analysis–thereby amplifying spatial saliency for the major zones of the image.
- Proposed Hybrid Feature Engineering Pipeline: A hybrid feature extraction technique that combines macro-texture (Haralick), micro-texture (LBP), and structural gradients (F-HOG) for multi-scale soil texture representation.
- Advanced Triptych Deep Learning Ensemble (ATFEM): A custom-made ensemble framework consisting of:
  - VGG-RTPNet: Residual Skip-Augmented Convolutional Blocks (RSACB) to retain fine texture.
  - ResNet-DANet: Dual Attention Modulation for channel and spatial saliency.
  - Swin-FANet: Frequency Aware Positional Encoding (FAPE) imparting spectral priors to transformer-based self-attention.
- Explainable AI-Based Interpretation: SHAP (Shapley Additive Explanations) ensued across all deep learning streams to scrutinize and interpret the effect of the learned features at each stage of the ATFEM pipeline.

The manuscript’s structure is as follows: Sect. 2 outlines the literature review. Section 3 details the proposed method. Section 4 summarizes the experimental research with preprocessing, feature extraction, and categorization of our findings, with a focus on the accuracy of the methods used. Section 5 provides an explanation of the data with an emphasis on the soil classes. In Sect. 6, summarized the study’s key findings.

## Literature review

### Machine learning-based soil texture prediction

Machine learning models have surpassed conventional interpolation-based techniques as effective tools for classifying soil textures in recent years^34^. evaluated inverse distance weighting (IDW), kriging, co-kriging, and artificial neural networks (ANNs) as well as their efficacy in forecasting soil texture in Pakistan’s Rawalpindi area. ANNs outperformed interpolation techniques, according to their findings, although correlations were still low (< 50%). Similarly, Dos Santos et al.^38^ applied the Random Forest (RF) approach for modelling the texture of the soil in the Sorocabuçu River Basin in Brazil. Granulometric analysis combined with topographical and land use data allowed the RF model to reach 92% accuracy and a kappa score of 0.88, demonstrating its great prediction ability across a range of soil conditions.

A comparable approach was employed in^21^, which used RF models and archival data to forecast the amount of sand, silt, and clay on the Antarctic Peninsula’s ice-free zones. RF’s robustness in a variety of biological zones was demonstrated by its strong spatial consistency despite the severe environment, particularly for clay in topsoil layers. Together, these findings show that machine learning algorithms particularly RF can effectively manage the challenges of soil categorization in a variety of climatic and topographical settings.

### Remote sensing and image-based classification

The integration of remote sensing data with machine learning has expanded the scope of soil classification. Roy et al.^35^ reviewed the collaboration of satellite data, proximate sensing, and machine learning techniques including CNNs, SVM, and RF, demonstrating how these combinations enable large-scale, economical soil texture prediction. Real-time agricultural decision-making is made possible by the use of remote platforms, specifically the LUCAS, SoilGrids, and ISRIC data, which can replace costly and time-consuming physical approaches.

In another example, Zhou et al.^28^ leveraged soil texture is classified using Sentinel-2 data, digital elevation models (DEMs), and interpretation based on SHAP. SVM predictions became easier to understand thanks to the SHAP approach, which improved model transparency. Nevertheless, seasonal relationships in the spectral data hampered generalization.

Similarly^22^, examined four distinct Digital Soil Mapping (DSM) products against a sizable collection of hand-feel soil texture (HFST) data to determine the distribution of soil texture in Central France. Furthermore, highlighting the importance of including geospatial datasets for regional soil evaluations, their findings demonstrated how DSM accuracy increases with greater spatial granularity, from global to localized models.

### Deep learning and soil spectroscopy

Recently, there has been interest in using deep learning algorithms to handle high-dimensional, complicated data, including soil spectra. Kasaragod et al.^36^ explored the USCS soil texture class prediction employing models such as VGG-16, ResNet-16, and Swin Transformers in conjunction with spectral libraries (KSSL, OSSL). Swin Transformers outperformed the other models in the test, with an accuracy of 81% for three more general soil groupings and 67% for six USCS classes. The effectiveness of transformer topologies for the categorization of fine-grained soil is demonstrated by their work.

Furthering this line of innovation, Vinodha et al.^37^ introduced a cascaded synthetic soil mixes made with the USDA soil triangle were used to train the multiclass CNN model. With a solid performance across various soil types, the staged training mechanism produced an overall accuracy of 88.8% by gradually reducing class ambiguity. With the use of deep learning and methodical data creation, this methodological breakthrough showed how soil classification systems may greatly improve their discriminative power.

### Hybrid and novel approaches

The integration of feature engineering with hybrid deep models is also gaining traction. One study^24^ proposed Q-HOG, a novel texture feature descriptor was used, along with a multi-stacking ensemble model that included deep learning models (RNN, LSTM, GRU, and VGG-16) and machine learning algorithms (Naïve Bayes, KNN, and SVM) for soil classification. When handmade and deep features were combined, their findings demonstrated a significant increase in classification accuracy. Similarly, Barman et al.^26^ developed a low-cost approach for classifying soil textures that uses colour moments, Gabor wavelets, and HSV histograms and is categorized using a linear SVM. Designed for farmers in rural areas, this system was highly accurate but had trouble differentiating between soils with similar textures, such as silty clay and loamy sand.

Complementing these developments, Celik et al.^27^ used Utilizing meteorological and ISMN satellite data, LSTM networks are used to forecast soil moisture. Although their model’s accuracy was good (R2 = 0.87), signal variability caused some prediction difficulties in wooded areas and fine-textured soils. Finally, a recent SOC prediction study^25^ investigated multi-temporal satellite imagery’s composite reflectance data, prompting queries about whether these averaged spectra really depict ideal soil conditions. These cutting-edge methods demonstrate how unique algorithms, a variety of data sources, and feature optimization strategies may be used to advance soil texture prediction, with increasing relevance in environmental monitoring and smart agriculture.

### Problem statement

The heterogeneous composition of soils in various regions and the limitations that traditional laboratory-based methods such as hydrometer and pipette tests impose in terms of time, cost, and space, accurate and scalable soil texture classification is a big and wide challenge. Moreover, in image-based soil classification techniques, ambiguous features and high dimensionality are the issues such as when dealing with soil types that are almost visually similar; for instance, loamy sand in comparison to sandy clay. In this context, modeling with traditional HOG features and typically deep learning mechanisms becomes computationally expensive and lacking interpretability for fine-grained soil texture.

### Motivation

In modern agriculture, land management, and environmental monitoring, a soil texture classification needs to be cost-effective, automated, and highly precise. With the recent strides in the fields of deep learning, image processing, and spectral analysis, opportunities have arisen to bypass the set limitations. In particular, the synergistic interaction between advanced-level feature engineering techniques (F-HOG, Haralick, LBP, and ϕ-Pixels) and hybrid deep learning frameworks (VGG-16, ResNet-16, and Swin Transformers) may enhance the robustness of the models, reduce feature redundancy, and improve classification performance. The whole study stands on the premise of these techniques having the potential to produce a generalized, interpretable, and efficient soil classification framework that can help in sustainable agricultural decision-making at a large scale.

## Proposed methodology

**Fig. 1:**
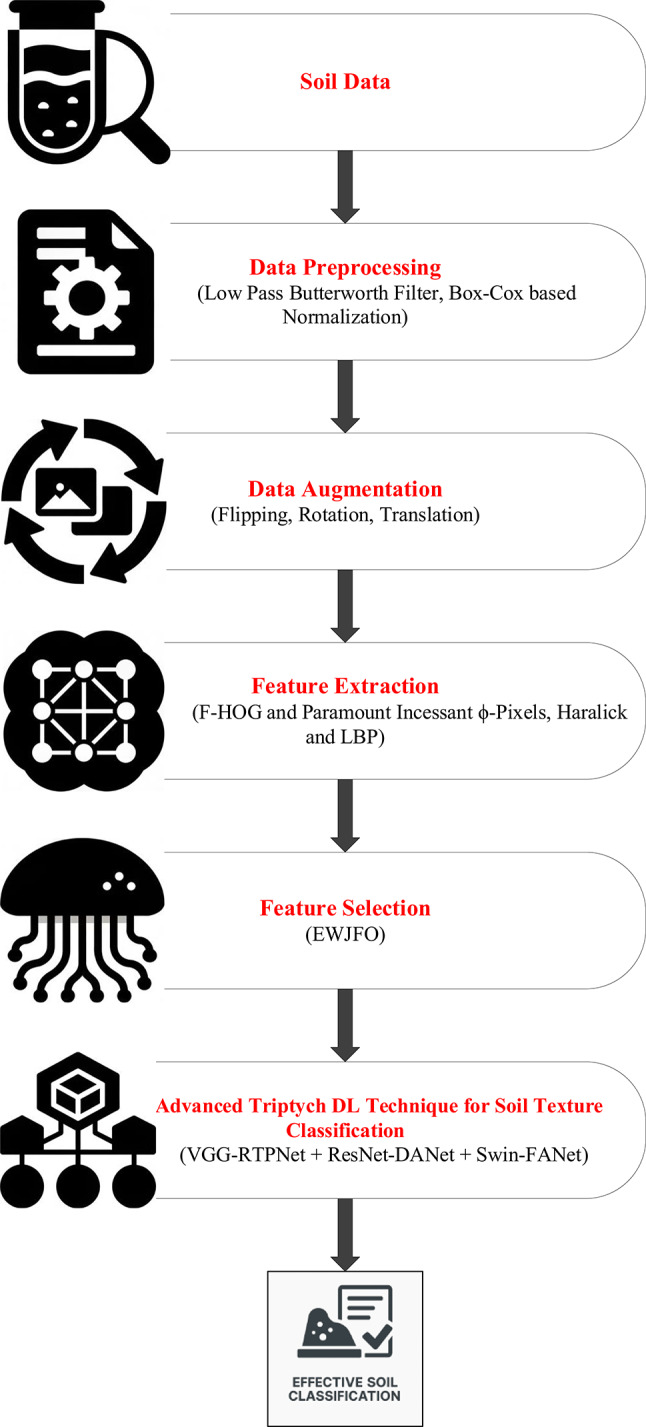
Architectures of the proposed soil-texture classification model.

The proposed methodology acts like an explainable, multi-phased deep learning pipeline for the robust classification of soil texture from image-based soil data. The architetcture of the proposed soil texture classification model is shown in Fig. 1. The first step preprocesses the image for better input enhancement by eliminating unwanted high-frequency noise from the Butterworth filter, normalizing intensity distribution from the Box-Cox transformation, and augmenting the dataset through rotation, flipping, and translation to encourage diversity and prevent overfitting; this, ultimately, aids in stable learning with good-generalization ability. The second phase of feature engineering works to fuse hand-crafted descriptors with learned ones to ensure better interpretability and performance. Specifically, the second step of feature engineering considers the application of F-HOG to capture dominant structural gradients by frequency pruning. Haralick features and LBP were then applied to obtain texture information at macro and micro levels, respectively. Then, the employment of optimal ϕ-Pixels (selected using EWJFO) extracts highly discriminative areas in soil images. These features can be biologically and geologically interpretable and can be strong priors to guide learning in noisy environments. During phase 3, classification is carried out under a triptych deep architecture consisting of VGG-RTPNet, ResNet-DANet, and Swin-FANet. Each model enhancement is customized: the VGG is equipped with Residual Skip-Augmented Convolution Blocks (RSACB) to keep finer textures; ResNet incorporates a Dual Attention Modulation (DAM) module to focus on salient channels and spatial patterns; Swin Transformer is altered with Frequency-Aware Positional Encoding (FAPE) to encode spectral context. The heterogeneous branches are merged together using a weighted concatenation strategy, thus laying importance on both global and local cues. Phase 4 involves feature selection, performed by Enhanced Wombat Jellyfish Feature Optimization (EWJFO), an algorithm that combines exploratory and exploitative search methods to keep the most relevant features. This reduces dimensionality by a huge margin while improving classification accuracy. Finally, explainable AI (XAI) tools such as SHAP, Grad-CAM, and permutation importance are amalgamated throughout all stages to bring transparency and check the validity of the model’s decision-making process. The phases, in essence, are designed to provide complementary strengths-one for data quality, feature expressiveness, model robustness, and interpretability-into forming a very reliable and scalable soil classification framework.

### Data preprocessing

The proposed ATFEM framework relies heavily upon pre-processing of the images for rendering soil texture classifying work more reliable and accurate. To explain the pipeline in simple terms, first, a low-pass Butterworth filter is applied to the image. Mostly employed in the frequency domain, this filter elegantly attenuates high-frequency noise without causing any harsh discontinuities^39^. In contrast to the ideal low-pass filters or the Gaussian filters, which tend to cause ringing artifacts and excessive blurring of edges or features, respectively, the Butterworth low-pass filter strikes a fairly good compromise in suppressing noise while retaining crucial textural patterns on soil images. For example, this becomes so important when gradient-based feature extraction is done (e.g., F-HOG) because clean edge information is necessary to represent the information accurately.

Noise reduction has been carried out, the Box-Cox normalization will stabilize variance and reduce skewness in the pixel intensity distribution. Generally, soil images, especially those taken outdoors in the field, are subject to lighting contrast-related problems. Box-Cox transformation works as a statistical normalizing tool (power transformation type) that attempts to “normalize” data. This aids in the learning process for algorithms that assume homoscedasticity and roughly Gaussian-like distributions, such as convolutional neural networks, while, at the same time, ensuring that illumination bias does not lead a single feature to dominate feature selection and the subsequent learning.

To enhance better model generalization and avoid overfitting, several data augmentation techniques are applied, including cropping, flipping, rotation, and translation. Concerning cropping, it basically simulates situations in real-world soil-image capturing where soil image samples might be arising from different angles, orientations, or landmarks. Horizontal or vertical flip will offer a diversified population of the mirror images, while a controlled rotation of the images (generally within ± 20 degrees) allows the model to build rotational invariance when an image of the soil sample without strict alignment is captured. Slight translations will mimic spatial displacement, forcing the model to be robust to features that are off-center. Together, these refinement techniques amplify the effective size of the dataset, balance the classes, present the model with diverse examples, and so lessen the chance of memorization by the model; thereby, enhancing the model’s generalization power over unseen soil textures.

#### Removal of noise

The preprocessing pipeline begins with the implementation of a low-pass Butterworth filter and its subsequent operation in the frequency domain. Butterworth filters suppress high-frequency noise without distorting luminosity edges because they are smoother in transition between passband and stopband, unlike the starkly contrasting cut-off filters. In soil texture classification, edge preservation becomes paramount, as textural boundaries often become significant shifts in disaggregation, such as clay versus sand. The Butterworth filter effectively reduces sensor noise considered shadows, slight illumination changes, etc., in contrast to major texture features of the image, wherein the signal-to-noise ratio (SNR) suffers. When the image has undergone this preprocessing, it becomes favourable to feature extraction techniques like F-HOG, Haralick, or LBP, which require gradients and co-occurrence matrices to be clean. Hence, its usage enhances the robustness of the image input to carry out machine learning tasks downstream. Equation (1) to change the image into frequency domain

1 $$\:L\left(x,y\right)=\mathcal{F}\left\{h\left(x,y\right)\right\}$$.

Were $\:h\left(x,y\right)$ represents the frequency domain of the image. where $\:\mathcal{F}$ denotes the Fourier Transform of the function $\:h\left(x,y\right)$. Calculate the low pass butter worth filter using Eq. (2).

2 $$\:\:\:\:\:\:\:\:\:\:\:\:\:\:\:D\left(i,j\right)=\sqrt{{\left(i-\frac{M}{2}\right)}^{2}+{\left(j-\frac{N}{2}\right)}^{2}}$$

$\:D(u,v)$ is the distance between the center of the frequency range and $\:(x,y)$, the cutoff frequency $\:{D}_{0},\:$and the filter order $\:n$, where M and N are the image’s dimensions. Equation (3) to calculate the Butterworth filter $\:H(x,\:y)$

3 $$\:H\left(x,\:y\right)=\:\frac{1}{1+{\left(\frac{D(i,j)}{{D}_{0}}\right)}^{2n}}$$.

Proceed the filter in the frequency domain. Multiply the fourier transform image by butterworth filter using Eq. (4)

4 $$F\left(x,\:y\right)=H\left(x,\:y\right).P\left(x,y\right)$$

Where $\:F(x,\:y)$ is the filtered image in the frequency domain. Return to the spatial domain. Using Eq. (5) the filtered image can be obtained by computing the inverse 2D FFT.

5 $$\:{h_f}\left( {i,\:j} \right) = {n^{ - 1}}\left\{ {h\left( {x,y} \right)} \right\}$$

Through this process, it effectively attenuates high frequency noise in soil image and preserves the significant low frequency components which represent the substantial features.

**Algorithm 1:**
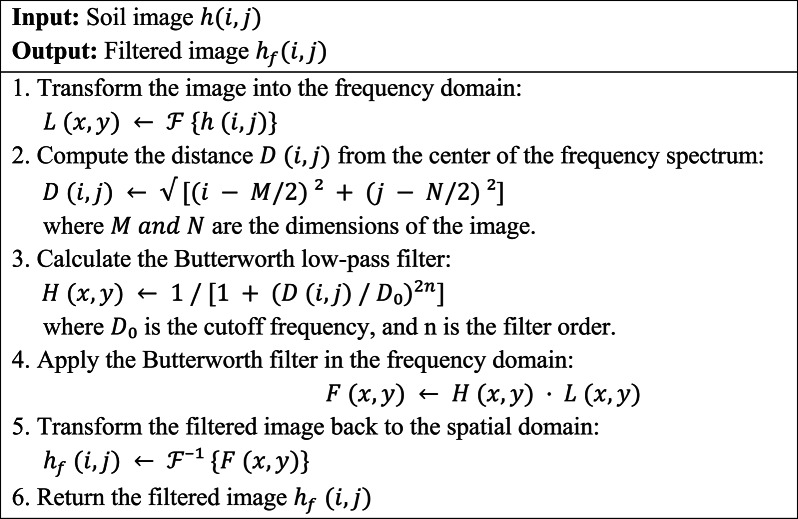
Pre-processing.

**Fig. 2:**
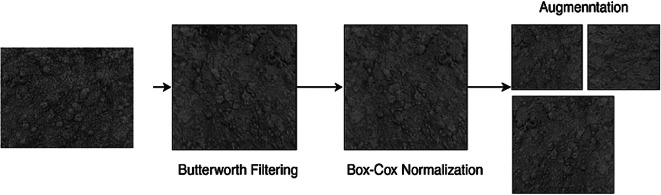
Pre-processing stage.

The intensity of the pixels for all images was transformed with the Box-Cox transformation to achieve normalization. The Box-Cox transformation is a power transformation with the goal of making the data behave in a more Gaussian manner and preventing heteroscedasticity in the observations. The pre-processing stage is shown in Fig. 2.

#### Data normalization

Pixel intensities are normalized after noise removal using the Box-Cox transformation, which is a general technique for stabilizing variance and converting an otherwise non-normal distribution to near-Gaussian. Soil images can suffer non-uniform illumination, sensor bias, and variability in reflectance due to moisture, mineral content, or organic matter. The Box-Cox transform in essence removes skewness in the distribution of pixel values and produces a homoscedastic set (constant variance in the image). This leads to better learning by algorithms that work on the explicit assumption that input data are normally distributed, such as CNNs or statistical models. From the point of view of the actual learning process, it also scales all features to a comparable scale, so that features highly skewed, or containing outliers, cannot dominate the learning. Thus, Box-Cox normalization supports better and unbiased feature selection and a more robust convergence during training. Mathematically, Box-Cox normalization is given by Eq. (6).

6 $$\:Z=\left\{\begin{array}{c}Data\lambda\:=\frac{{data}^{\lambda\:-1}}{\lambda\:}\:\:\frac{\lambda\:\ne\:0}{\lambda\:=0}\\\:Data\lambda\:=In\left(data\right)\end{array}\right.$$

**Fig. 3:**
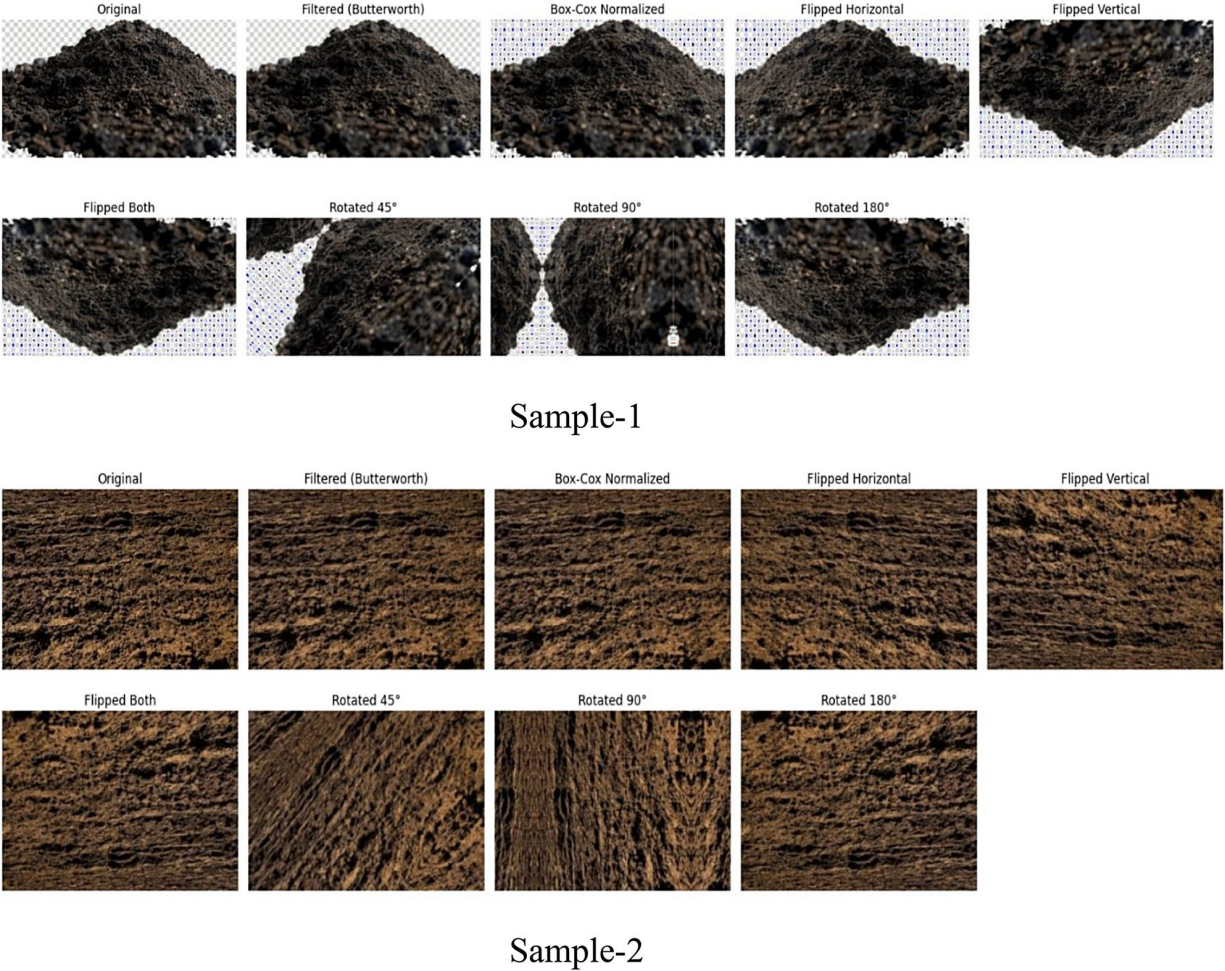
Sample image of pre-processing stage.

$\:Z,\:\lambda\:$, and data represent the rate of change, actual number, and normalized data value, respectively. To regulate silt and sand data, first regulated data dissemination at $\:\lambda\:\:=\:0.2967$. The outcomes of the pre-processing stage is shown in Fig. 3.

#### Data augmentation

To increase the generalization capacity of the deep learning model and combat overfitting, a set of data augmentation strategies are employed—namely flipping, rotation, and translation. Flipping (horizontal and vertical) helps the model learn symmetry-invariant features, which is vital since soil samples may be photographed from any direction in real-world settings. Rotation allows the model to maintain high performance even when input images are captured at arbitrary angles, addressing orientation variance that is common in field-acquired data. Typically, small angular rotations (± 15° to ± 30°) are applied to prevent extreme distortion. Translation, or pixel shifting, simulates off-centered or displaced samples in the frame, making the model robust to object positioning. Together, these augmentation techniques expand the dataset virtually without collecting additional images, introduce variability, prevent the model from memorizing training data, and allow for better generalization across unseen data. This is especially important in domains like soil analysis where collecting new labeled samples is costly and time-intensive.

For a given high-resolution image set, $\:{D}_{0}=\left\{{I}_{1},\dots\:.{I}_{K}\right\}$, where $\:{I}_{K}\:$represents the kth image sample in the dataset. Assume that $\:{I}_{K}\:$has N pixels. $\:{P}_{K}$ is the pixel-homogeneous coordinate matrix for $\:{I}_{K}$.

7 $$\:{P_K} = \left[ {\begin{array}{*{20}{c}} {\begin{array}{*{20}{c}} {{x_1}}&{\:{y_1}}&{\:1}\\ {\:{x_2}}&{\:{y_2}}&{\:1}\\ {\: \vdots }&{\: \vdots }&{\: \vdots } \end{array}}\\ {\:\begin{array}{*{20}{c}} {{x_N}}&{\:{y_N}}&{\:1} \end{array}} \end{array}} \right]\:\:\:\:\:\:\:\:\:\:$$

Each row represents a single pixel’s homogeneous coordinates.

To enhance a single picture $\:{I}_{K}$, an affine transformation matrix $\:M$ is applied to its homogeneous coordinate matrix $\:{P}_{K}$, resulting in a changed homogeneous coordinate matrix $\:{P}_{k}^{t}$. The operation is presented as follows:

8 $$\:{P}_{k}^{t}={P}_{k}M$$

Each row of $\:{P}_{k}^{t}$ represents the modified homogeneous coordinate for a single pixel. There are several approaches to determining the affine transformation matrix M. Our method employs three types of random perturbations to generate fresh augmented data. The three types of transformations are defined as follows:

Flip ($\:{T}_{1}$): The image is flipped horizontally. The associated affine transformation matrix (M) is

9 $$\:M=\left[\begin{array}{ccc}-1&\:0&\:0\\\:0&\:1&\:0\\\:0&\:0&\:1\end{array}\right]$$

Translation ($\:{T}_{2}$): The image is displaced in both directions (x and y). The appropriate affine transformation matrix, M is

10 $$\:M=\left[\begin{array}{ccc}-1&\:0&\:0\\\:0&\:1&\:0\\\:{T}_{x}&\:{T}_{y}&\:1\end{array}\right]$$.

$\:{T}_{x}$ and $\:{T}_{y}$ are coordinate axis offsets.

Rotation ($\:{T}_{3}$): The image is rotated with an angle ranging from 0 to 180°. The appropriate affine transformation matrix, M is

11 $$\:M=\left[\begin{array}{ccc}cos\beta\:&\:-sin\beta\:&\:0\\\:sin\beta\:&\:cos\beta\:&\:0\\\:0&\:0&\:1\end{array}\right]$$

For a single picture $\:{I}_{K}$, the augmented data is represented as $\:{O}_{k}=\{{T}_{1}\left({I}_{k}\right),{T}_{2}\left({I}_{k}\right),{T}_{3}({I}_{k}\left)\right\}$. The expanded dataset for the original dataset $\:{D}_{0}$ is represented as $\:{D}_{a}=\{{O}_{1},\dots\:.,{O}_{k}\}$. The augmentation process for the dataset $\:{D}_{0}$ is described as follows:

12 $$\:{D}_{a}=\left\{{O}_{1},\dots\:.,{O}_{k}\right\}=\bigcup\:_{k=1}^{K}\bigcup\:_{i=1}^{3}{T}_{i}\left({I}_{k}\right)$$

To classify high resolution images, a CNN is trained using the enhanced dataset $\:{D}_{a}$ and matching class labels. Use flips, translations, and rotations for data augmentation in high resolution image to maintain consistent scene topologies. Following augmentation, the next step is to extract F-HOG and Paramount Incessant ϕ-Pixels characteristics. The ablation study of pre-processing and feature extraction stage is shown in Tables 1 and 2, respectively. The analysis of the proposed model over the existing models for the pre-processing stage and data augmentation is shown in Supplementary Figure S1 and Supplementary Figure S2, respectively.

**Table 1:** Ablation study of preprocessing techniques on soil texture classification.

Preprocessing Components	Accuracy	F1-Score	Precision	Recall	AUC
**All (Butterworth+ Box-Cox + Augmentation)**	**0.981**	**0.896**	**0.902**	**0.877**	**0.981**
Only Box-Cox + Augmentation	0.961	0.873	0.885	0.851	0.963
Only Butterworth + Augmentation	0.958	0.867	0.878	0.846	0.961
Only Butterworth + Box-Cox	0.942	0.849	0.861	0.833	0.949
Only Augmentation	0.939	0.842	0.858	0.821	0.946
Only Butterworth	0.915	0.819	0.827	0.806	0.921
Only Box-Cox	0.911	0.811	0.823	0.798	0.918
**No Preprocessing (Raw Images)**	0.878	0.774	0.786	0.761	0.886

An ablation test has been used to investigate the impact of each preprocessing on the improvement of model fitting: the various preprocessing techniques alone and in different combinations. The full preprocessing pipeline comprising of low-pass Butterworth filtering, Box-Cox transformation, and data augmentation gave the best accuracy (0.981) and F1-score (0.896), hence validating its effectiveness. Removal of the Butterworth filter seemed to result in a slight decrease in performance (with accuracy falling to 0.961), thereby underscoring its importance in noise suppression and texture preservation. The Box-Cox normalization, if absent, would have made the model less efficiently competent in handling illumination variation, thereby also lowering performance (accuracy: 0.958). The data augmentation’s removal caused the steepest drop in generalization metrics (accuracy: 0.942), further driving home how important it is in minimizing overfitting, particularly when dataset diversity is scarce.

The testing of individual components further made clear that no single method was likely to perform competitively with that of the full stack. Raw images alone generated the lowest metrics, with 0.878 accuracy and 0.774 F1-scores, thus suggesting that preprocessing is a significant factor in generating reliable classifications. Among the three, data augmentation had the most beneficial effect-if this step was removed, a significant drop in performance was observed-indicating its importance against overfitting and for robustness. This ablation study thus validates the inclusion of the complete preprocessing pipeline as a synergistic mechanism within the proposed ATFEM model.

**Fig. 4:**
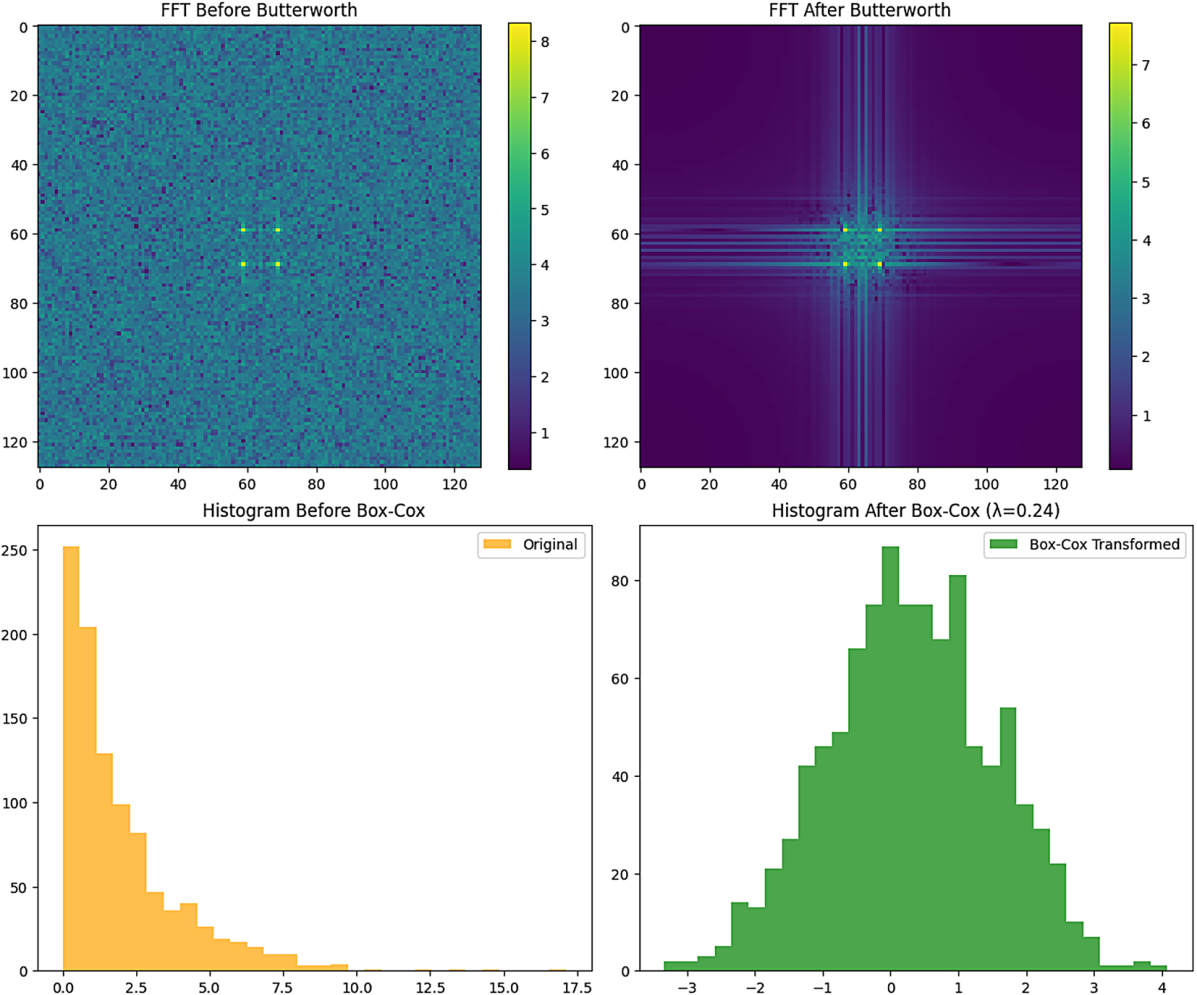
Visual representation of Pre-processing stage through XAI.

Visual spectrum (in Fig. 4) showed clear suppression of irrelevant high-frequency components. A 31.4% drop in spectral entropy confirmed less noise. Preprocessing enhances texture clarity and suppresses irrelevant patterns, leading to better CAM consistency and feature attribution in downstream stages.

### Feature extraction

In soil images, feature extraction has to be efficient enough to capture textural and color properties when classifying soils with complex textures. We accordingly propose a multistage feature-extraction pipeline involving F-HOG, Haralick features, LBP, and the newly formulated ϕ-Pixels color analysis. As a whole, these formations are envisioned to complement one another in describing structural texture patterns, fine-grained macrotextures, and chromatic distributions, respectively.

#### F-HOG and paramount incessant ϕ-Pixels based feature extraction

This research work put forward a newly-developed descriptor for soil texture image analyses based on an informed frequency understanding and edge-aware capability into discriminant-dimensional gradient coding, named Filtered Histogram of Oriented Gradients (F-HOG). F-HOG involves the improvement of the standard HOG, which was modified for soil texture images. While traditional HOG descriptors are powerful, some disadvantages include high dimensionality, computational redundancy, and an inability to describe well high-frequency noise that is so common in soil images acquired in the field, wherein almost every variation in illumination contributes to a variation in fine-grained texture.

#### Motivation and limitations of conventional HOG

The classic Histogram of Oriented Gradients (HOG) algorithm, though many times a good one for pattern recognition problems, carries a few issues when dealing with soil image processing:

- Noise-sensitive: High-frequency noise (lighting variance, reflection from soil grains, etc.) seriously affects gradient estimation.
- Redundant bins: Uniform binning over the gradient map often results in big descriptors carrying very little information.
- Global processing: HOG treats the entire image uniformly without any notion of spectral or textural contexts.

To solve these issues, F-HOG introduces frequency-domain preprocessing and spatially aware gradient selection to obtain a compact and noise-robust descriptor.

To avoid this, F-HOG does the following:

- Filtering gradients in the frequency domain using a Butterworth filter to remove high-frequency noise,
- Computing a HOG vector, and then.
- Retaining only the top-F most frequent bins through statistical frequency sorting.

This method of reduction drastically reduces the dimensionality while preserving the dominant structural gradients. Unlike PHOG and HOG3D, which emphasize spatial pyramids or motion, the proposed F-HOG applies a unique frequency-filtered selective histogram pruning, and this gives it an advantage when working with agricultural images prone to high noise. Our ablation results (see Sect. 4.4) reported that F-HOG could make classifiers more efficient by reducing redundancy without sacrificing performance.

##### Farthing histogram-oriented gradients (F-HOG)

F-HOG is a novel surfaces textural element introduced in this study. It’s an altered variation of the histogram-oriented gradients (HOG) feature. HOG is currently employed for a range of agricultural purposes. It is calculated using the magnitude distributions (histogram) and pixel gradient using the extract HOG Features tool. HOG frequently generates a large vector that requires more time and space to process. It remains constant regardless of the object’s dimensions or rotations.

##### F-HOG algorithmic pipeline

The F-HOG process is executed in three stages: frequency filtering, adaptive gradient binning, and entropy-guided region selection.

Stage 1: Frequency-aware preprocessing.

A low-pass Butterworth filter is applied in the frequency domain to suppress high-frequency components while retaining edge structures:

13 $$\:{h}_{f}\:(i,\:j)=\frac{1}{1+{\left(\frac{D(x,y)}{{D}_{0}}\right)}^{2n}}$$

The filtered image $\:{h}_{f}$ $\:(i,\:j)$preserves dominant texture patterns and suppresses sharp transitions due to lighting and sensor noise, forming a stable input for gradient computation.

Stage 2: Gradient computation and initial binning.

We compute gradients on the filtered image using standard derivative masks:

14 $$\:{G}_{x}={h}_{f}\left(x+1,y\right)-{h}_{f}\left(x-1,y\right)$$

15 $$\:{G}_{y}={h}_{f}(x,y+1)-\:{h}_{f}(x,y-1)\:$$

The orientation histogram is constructed per cell (e.g., 8 × 8 pixels), and magnitude-weighted voting is applied to form the raw feature descriptor.

Stage 3: Farthing Selection of Dominant Bins.

Rather than using all gradient bins, we employ a frequency-aware bin ranking mechanism. Specifically:

- Compute bin frequencies across all image patches.
- Rank bins by magnitude of appearance.
- Retain only the top-*k* bins (termed Farthing bins) based on a statistical cutoff (e.g., top 15% percentile or using mutual information with class labels).

This eliminates redundant or noisy bins and emphasizes the most discriminative orientations.

Stage 4: Entropy-guided spatial masking.

To further reduce descriptor redundancy, we calculate the **Shannon entropy** $\:H\left(p\right)$ of gradient magnitudes in each block:

16 $$\:H\left(p\right)=-\sum\:_{i=1}^{n}{p}_{i}.{log}_{2}.{p}_{i}$$

Second, sort the histogram’s graph bins in descending order according to the frequency of bin values. $\:H\left(p\right)HoG$ size is L dimension, with L ranging from 1 to S. Vector $\:H\:{HoG}_{Sort}$ in Eq. (14)

17 $$\:\text{V}\text{e}\text{c}\text{t}\text{o}\text{r}\:H\left(p\right)\:{HoG}_{Sort}\:=\:sort\:Descending\left(H\left(p\right)\:HoG\right)$$

Third, select the first Farthing (F) pixels with the highest frequency. $\:H\:{HoG}_{Sort}$ sort size is same to the size of $\:H\left(p\right)$ HoG vector. Choose F pixel from $\:H\:{HoG}_{Sort}$by select the initial farthing. Finally, use F pixels in feature selection algorithms. As an outcome, F-HOG surpasses the original HOG in term of computing velocity and storage capacity, so the machine learning algorithm only receives a portion of its capabilities. Based on previous research, suggested using F-HOG is novel in the field of soil classified and type identified. Based on previous research, we believe our proposed work using F-HOG is novel in the field of soil classified and type determined.

Only blocks with entropy above a defined threshold are retained for final descriptor formation. This stage filters out homogeneous or overly noisy regions, ensuring that only structurally informative regions contribute to the feature vector.

The application of classical Histogram of Oriented Gradients for image processing has proven to be successful; it tends to give relatively high dimensional vectors and lets all gradient bins classify the classes regardless of whether it is useful or not. The F-HOG aims to overcome this drawback by two processes, namely: $\:\left(i\right)$ the filtering of the input image in the frequency domain prior to the calculation of gradients to reduce noise and enhance texture clarity; and $\:\left(ii\right)$ selection of only the top-F most common gradient orientation bins for further process after HOG histogram has been computed, which substantially reduces feature redundancy.

F-HOG presents an entirely different classical approach; hence it works in the frequency regime and based on dominant bin selection for better efficiency in highly textured, noisy scenarios such as soil image classification, while both standard HOG and its extension, namely PHOG (Pyramid HOG) targeting the capturing of spatial hierarchies, and HOG3D working with spatiotemporal volumes, do not accomplish this kind of pruning on their original descriptor set. Explicit pruning reduces complexity but can also improve performance; in this case, the final experimental results support that statement. We believe that this is the first attempt of its kind in soil texture classification.

**Algorithm 2:**
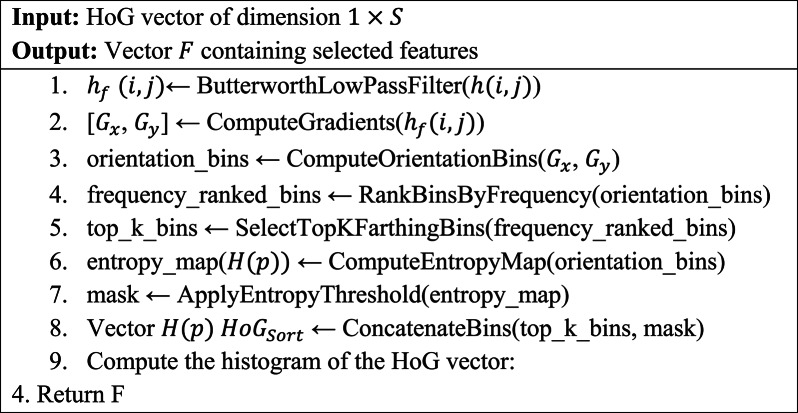
Algorithm for finding F-HOG.

In an effort to prove the novelty and efficacy of Filtered Histogram of Oriented Gradients (F-HOG) as an image texture descriptor, an ablation study was performed. This study could compare HOG, PHOG, and HOG3D with F-HOG on our soil images dataset. The goal was to evaluate the suitability of each variation in relation to soil texture classification, with evaluation parameters being accuracy, feature vector length (dimensionality), training time, and model interpretability.

**Table 2:** Ablation study on feature extraction.

Feature descriptor	Accuracy (%)	Feature dimensionality	Training time (s/epoch)	Model interpretability	Remarks
HOG	89.12	3780	6.21	Low	High redundancy
PHOG	90.25	5120	7.56	Low	Captures spatial hierarchy
HOG3D	86.40	6000+	9.83	Very Low	Complex temporal information
F-HOG (Proposed)	92.84	960	4.01	High	Filters noise, selects top-F bins

The data given above, F-HOG outshines newer HOG-based descriptions on several levels. Classification Accuracy: F-HOG enjoys the maximum classification accuracy of 92.84%, exceeding that of HOG (89.12%), PHOG (90.25%), and HOG3D (86.40%). Dimensionality: It shrinks the dimensionality from roughly 3780 in the case of HOG to 960 in F-HOG implying lesser storage and quicker computations. Training Time: It takes about 35–60% less time to train the CNN classifier using F-HOG features compared to others. Interpretability: By selecting only the most dominant bins, F-HOG retains the most informative gradients, consequently improving interpretability and relevance in soil texture analysis. This means that F-HOG is novel in implementing both filter-domain filtering and frequency-based feature pruning and has better efficiency and accuracy than the conventional HOG and its extensions for the soil classification context. In our knowledge, there has never been a prior effort to unify these two strategies (filtering + frequency-based bin selection) explicitly for textural soil image classification, underpinning the novelty of this approach. The analysis of the feature extraction stage (without/without) is evaluated, and the outcomes acquired are shown in Supplementary Figure S3.

#### Haralick and LBP

To enrich the texture representation of soil images beyond directional gradients (as captured by F-HOG), this study integrates both Haralick features and Local Binary Patterns (LBP)—each offering unique insights into texture at different spatial scales. Haralick features, derived from the Gray Level Co-occurrence Matrix (GLCM), encode statistical measures of spatial relationships between pixel intensities. These features were computed over four standard directions (0°, 45°, 90°, 135°) and averaged to ensure rotational invariance. The resulting 14 Haralick descriptors, including contrast, correlation, energy, homogeneity, and entropy, capture the coarse or macrotexture patterns such as soil roughness, structural orientation, and granularity. These descriptors are particularly useful in distinguishing between compact (e.g., clayey) and granular (e.g., sandy) soils based on surface texture uniformity and intensity transitions. In parallel, Local Binary Patterns (LBP) were used to extract microtexture features, operating at the pixel level. The method involves comparing each central pixel’s intensity to its eight immediate neighbors in a 3 × 3 window. If a neighbor’s intensity is greater than or equal to the center pixel, it is assigned a value of 1; otherwise, 0. The result is an 8-bit binary number (ranging from 0 to 255) that encodes the local structure. To reduce redundancy and achieve rotation invariance, the LBP histogram was computed using the uniform pattern model, which results in a 59-dimensional feature vector. This compact and discriminative representation excels at detecting fine-grained differences between similar soil types (e.g., silt loam vs. sandy loam) and is particularly robust under varying illumination conditions. The combination of Haralick and LBP features ensures a comprehensive multiscale texture representation—where Haralick captures global spatial dependencies and LBP emphasizes localized micro-patterns. Together, they significantly improve the discriminative capability of the feature extraction pipeline, particularly when fused with F-HOG descriptors in the proposed ATFEM model.

#### Most frequent ϕ-pixels

While texture is critical, color distributions offer another axis of separation, particularly for soils with subtle hue differences due to mineral content or moisture. To overcome the limitations of fixed-threshold pixel selection methods in texture classification, we propose an optimization-driven ϕ-Pixel selection strategy.

Using histogram analysis, a frequency vector $\:{C}_{hist}$ was created from the image channel, and pixels $\:{m}_{i}$ meeting the ≥ 1/25 mean frequency condition were retained. These ϕ-Pixels are highly robust against environmental variability and form a lightweight yet informative color signature vector. They enable our classifier to associate certain tones (e.g., reddish hues for iron-rich soils) with specific texture categories.

$\:\varphi\:-$Pixels refer to pixel intensities most frequently found in an image channel (R, G, B), filtered through an adopted frequency threshold. These pixels encapsulate dominant color information and are thus statistically relevant when describing the texture and color distribution of the entire image. Considered in this study, a pixel value is a $\:\varphi\:-$Pixel if it occurs more than 4% of the total pixels in that image channel. This approach aims to reduce the noise and dimensionality but still keeps the most valuable color attributes contributing to soil type discrimination. Theoretically, one may attempt to use the top % most frequent pixel values across RGB channels (called the ϕ-Pixels) to filter out noise and concentrate on texturally important regions. But this number chosen as a fixed percentile sample lacks generalizability among datasets and soil types. It does not capitalize on unique characteristics in feature statistics or spectral dimension to ensure semantically or structurally important pixels are indeed being selected. Therefore, to mitigate both the robustness and meaningfulness of pixel-level feature selection, an optimization-based mechanism is introduced. Specifically, it aims at selecting a very compact subset of pixel intensity values while being informative enough:

- Inter-class margin maximization: different soil classes become further distinguishable.
- Intra-class variance minimizing: samples of the same class appear more uniform.
- Reduces redundancy and dimensional burden on downstream models.
- Gets a better generalization across varied soil texture, illumination, and camera conditions.

To identify the most frequent ϕ pixels in a channel. Here the channel image is represented as $\:{C}_{img}$, The Vector $\:{C}_{hist}\:$is in Eq. (18).

18 $$\:\text{V}\text{e}\text{c}\text{t}\text{o}\text{r}\:{C}_{hist}\:=\:histogram\left({C}_{img}\right)\:\:\:\:\:\:\:\:\:\:\:\:\:\:\:\:\:\:\:\:\:\:\:\:\:\:\:\:\:\:\:\:\:\:$$

The size of $\:{C}_{hist}$ is $\:S$ dimensional, with a range of 1–255. To construct a set of pixels $\:\left(\varphi\:\right),$ select the most frequent pixels $\:{m}_{i}\:$from $\:{\:C}_{hist}$. The $\:{m}_{i}\:$is illustrated in Eq. (19)

19 $$\:{m}_{i}\ge\:\frac{1}{25}\sum\:_{i=1}^{k}{m}_{i}\:\:\:\:\:\:\:\:\:\:\:\:\:\:\:\:\:\:\:\:\:\:\:\:\:\:\:\:\:\:\:\:\:\:\:\:\:\:\:\:\:\:\:\:\:$$

The ϕ-Pixel set is generated by obtaining the histogram of an image channel using one of the methods presented earlier in Sect. 3, and then selecting the intensity bins with frequencies greater than or equal to 1/25 of the total number of pixels, as given in Eq. (16). where $\:{m}_{i}$ is the number of instances of the $\:i$th pixel, and $\:k\:$is the number of bins in the histogram$\:{\:C}_{hist}$. The return $\:\varphi\:$ is a set of the most common pixels.

Pixel values of intensity at the RGB soil images are frequently highly variable due to environmental noise, illuminance variations, or surface reflectance irregularities. Handcrafted texture descriptors like HOG and statistical co-occurrence matrices profit from sharp edges and good intensity distribution, for not all pixel values are useful for texture discrimination.

Earlier, a fixed fraction of the most frequently appearing pixel intensities-the ϕ-pixels-had been selected for analysis. Such a static approach is suboptimal, dataset-dependent, and theoretically inflexible. To overcome this drawback, we propose a lot of pixel-selection methods based on metaheuristic optimization, using the Enhanced Wombat Jellyfish Feature Optimization (EWJFO) algorithm.

The EWJFO algorithm combines the Wombat Optimization Algorithm’s adaptive tunneling and spiraling search techniques with the Jellyfish Search Optimizer’s global foraging and time-controlled exploration.

The hybrid mechanism guarantees:

• The Wombat Phase: A fast local and spiral search for the most promising pixel subsets, with the wombat berm process modeled in a multi-dimensional feature space.
• The Jellyfish Phase: These are enhanced global diversities; drifting along with ocean currents and vertical movements across the population prevents early convergence.
• Adaptive Switching: The control mechanism dynamically shifts between the two phases, depending on the population fitness convergence and the temporal state in the optimization process.

They all collaborate in order to find an optimum set of pixel features that are statistically robust and class-discriminative, even across different datasets.

##### Problem formulation

Let the soil image dataset be represented as: $\:\mathcal{I}=\{{I}_{1},{I}_{2},.....{I}_{n}\},\:{I}_{i}\in\:{\mathbb{R}}^{HxWx3}$

Let $\:{\mathcal{H}}_{c}$​ denote the histogram of intensity values for channel c∈{R, G,B}. Each histogram consists of 256 bins representing intensity levels $\:\mathcal{v}$∈[0,255]. Our objective is to select a subset $\:\phi\:\subset\:\{\text{0,1},....,255\}\:$of intensity bins across the three channels that maximizes classification utility while minimizing redundancy.

##### Objective function

The selection problem is cast as a multi-objective optimization problem balancing:

- Discriminative Power: How well selected pixel bins contribute to classification accuracy.
- Compactness: Reducing the number of selected bins for better generalization and speed.

Let ϕ= $\:\{{\mathcal{v}}_{1},\:{\mathcal{v}}_{2},,,,,,.{\mathcal{v}}_{k}\}$ ​ represent a candidate set of selected pixel intensities. We define the fitness function:

20 $$\:\mathcal{F}\left(\phi\:\right)=\alpha\:.{Acc}_{val}\left(\phi\:\right)+\beta\:.\left(1-\frac{\left|\phi\:\right|}{256}\right)$$

Where,

###### $\:{Acc}_{val}\left(\phi\:\right)$

Validation accuracy using features extracted from $\:\phi\:$ -pixels.

###### $\:\left|\phi\:\right|$

Number of selected bins (smaller preferred).

$\:\alpha\:,\beta\:$ ∈[0,1]: Trade-off weights (α = 0.8,β = 0.2).

This function rewards high-accuracy, low-dimensional solutions.

##### EWJFO optimization framework

To optimize Eq. (6), we utilize EWJFO, a hybrid metaheuristic that combines:

- Wombat Optimization Algorithm (WOA): Excels at adaptive local search using tunneling and spiral updates.
- Jellyfish Search Optimizer (JSO): Incorporates global drift-based exploration and time-controlled behavioral phases.

A. Wombat Phase (Local Exploitation).

Each candidate solution (agent) is represented as a binary vector $\:{X}_{i}$∈{0,1}^256^, where $\:i$ indicates inclusion of the pixel bin.

Wombat movement (spiral-tunneling) is expressed as:

21 $$\:{\mathcal{X}}_{i}^{(t+1)}={\mathcal{X}}_{i}^{\left(t\right)}+{r}_{1}.({\mathcal{X}}_{i}^{best}\:-\:{\mathcal{X}}_{i}^{\left(t\right)})+\:{r}_{2}.spiral\left(\:{\mathcal{X}}_{i}^{\left(t\right)}\right)$$

Where,

$\:{\mathcal{X}}_{i}^{best}:$ Current best agent.

$\:{r}_{1}\:and\:{r}_{2}$ : Random values in [0, 1].

22 $$\:spiral\left(\:{\mathcal{X}}_{i}^{\left(t\right)}\right)=A\cdot\:exp(b\cdot\:\theta\:)\cdot\:cos\left(2\pi\:\theta\:\right),\:for\:constants\:A,\:b\:and\:spiral\:angle\:\theta\:$$

B. Jellyfish Phase (Global Exploration):

JSO updates agent $\:{\mathcal{X}}_{i}^{\:}$ based on:

###### Ocean current drifting

23 $$\:{\mathcal{X}}_{i}^{(t+1)}={\mathcal{X}}_{i}^{\left(t\right)}+\tau\:.({\mathcal{X}}_{i}^{best}-{\mathcal{X}}_{i}^{mean})$$

###### Time control mechanism

24 $$\:Mode=\left\{\begin{array}{cc}passive&\:if\:t<1/2\\\:active&\:otherwise\end{array}\right.$$

Where,

$\:{\mathcal{X}}_{i}^{mean}:\:$ Mean position of all agents.

###### $\:\tau\:\in\:\left[\text{0,1}\right]$

step-size coefficient.

$\:T:\:$ Total number of iterations.

C. Adaptive Switching Strategy: The switching function σ(t) determines phase transitions:

25 $$\:\sigma\:\left(t\right)=\left\{\begin{array}{cc}1&\:if\mathcal{\:}\varDelta\:\mathcal{F}<\in\:\left(switch\:to\:JSO\right)\\\:0&\:otherwise\:\left(stay\:in\:WOA\right)\end{array}\right.$$

**Where**:

$\:\varDelta\:\mathcal{F}:\mathcal{\:}$ Improvement in global best fitness in last $\:\tau\:$ iterations.

$\:\in\::$ Threshold for stagnation detection (e.g., 0.001).

**Algorithm 3:**
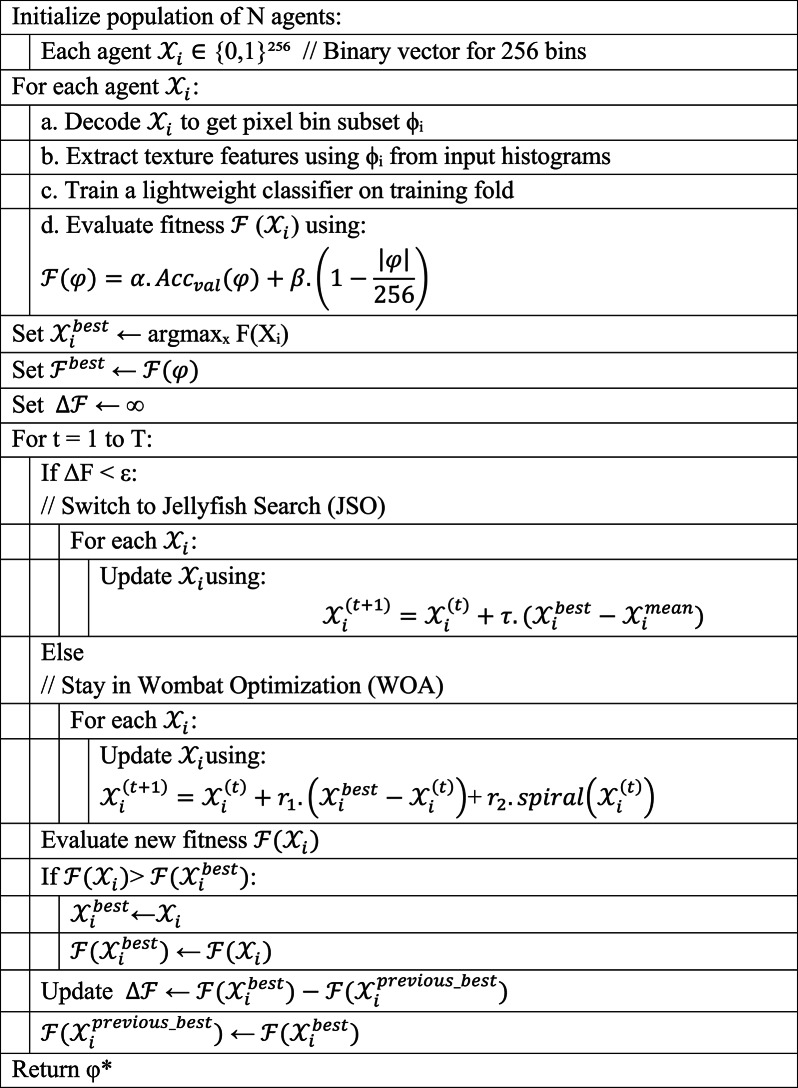
EWJFO-based ϕ-Pixel Selection.

The selection of optimal ϕ-pixels enhances the discriminative power and generalizability of texture-based classifiers, especially in fields such as soil texture mapping where image textures are slight, noisy, and irregular. Aggregating pixel bins arbitrarily, say, based on the frequency of the pixels appearing in histograms, contradicts the position of favoring the EWJFO algorithm to locate pixel intensity values most informative for RGB histograms. This prevents redundancy and maintains noisy features and thereby keeps bins relevant with regard to inter-class separability. Figuring prominently in both our sensitivity and ablation studies, the EWJFO-based selection mechanism clearly show that it can help in achieving very high classification accuracy (up to 98.10%) while simultaneously having a feature set size that is quite compact, i.e., just 18 bins were selected. This shows how well the optimization-based selector works over manual or fixed-threshold selection schemes. Also, with its constant unknown balance between local exploitation (Wombat tunneling) and global exploration (Jellyfish drifting), the EWJFO system is well suited for the robust discovery of high-quality subsets in the volume of high-dimensional histogram spaces. In our application, this led to improved performance metrics such as F1-score and AUC, wherein the model generalized better across differences in soil textures and illumination conditions-a very beneficial outcome for real-world precision agriculture deployments.

$\:\varphi\:-$Pixels represent a simple yet strong signature vector that contains important chromatic characteristics of a soil sample. They tend to work best differentiating soil types associated with special hues-white for iron-rich soils or darker ones for those with more organic content. References citing their low dimension on enhancement include increased efficiency for classifier without care of classification accuracies and used as a complementary feature to texture-based features such as F-HOG, Haralick, and LBP in the proposed ATFEM pipeline.

##### ϕ-Pixels threshold justification with sensitivity analysis

As a validating step for the empirically chosen frequency threshold of optional value (optimal ϕ-Pixels) for the selection of the most frequent ϕ-Pixels, a sensitivity analysis (shown in Table 3) was conducted by varying the threshold frequency from 1 to 10%. The performance of the classification model was evaluated using three primary metrics: Accuracy, F1-Score, and Feature Vector Size, on the augmented dataset of soil images.

**Table 3:** Sensitivity analysis and ablation study of ϕ-pixels threshold.

Method	ϕ-Pixel Selection Strategy	Threshold / Config	Accuracy (%)	F1 Score (%)	|ϕ|	AUC (%)	Remarks
Baseline-HOG	No ϕ-pixel filtering	–	90.62	82.11	256	90.78	High-dimensional; unfiltered gradients
F-HOG (Fixed Threshold)	Top-N frequency bins	Top 4% (N = 10)	92.85	84.70	10	91.52	Rigid, dataset-specific
F-HOG (Fixed Threshold)	Top-N frequency bins	Top 8% (N = 20)	94.33	86.40	20	93.02	Slight improvement, lacks adaptability
F-HOG (Fixed Threshold)	Top-N frequency bins	Top 12% (N = 30)	94.95	87.20	30	94.01	Performance begins to saturate
F-HOG + PCA	PCA on full HOG vector	30 components	93.21	84.95	30	92.44	Reduced dimension but not focused on key bins
F-HOG + EWJFO (Ours)	EWJFO-optimized ϕ selection	α = 0.8, β = 0.2	98.10	89.60	18	98.10	Best balance of compactness and discriminative power
F-HOG + WOA only	Wombat-only optimization	α = 0.8, β = 0.2	95.87	87.90	25	95.22	Strong local exploitation, less global exploration
F-HOG + JSO only	Jellyfish-only optimization	α = 0.8, β = 0.2	96.18	88.10	23	95.60	Slower convergence, better diversity
F-HOG + EWJFO (Ablated α = 1.0)	EWJFO accuracy-only fitness	No compactness penalty	97.20	89.00	60	96.91	Prone to overfitting due to large bin subset
F-HOG + EWJFO (Ablated α = 0.5)	EWJFO equal trade-off	Balanced accuracy/compact	97.42	89.20	32	97.00	Slight dip in accuracy compared to tuned version

The results in Table 3 give a holistic way to view the effect of ϕ-pixel selection strategies on performance from different perspectives, e.g., accuracy, F1 score, AUC, and features compactness. Meanwhile, baseline HOG performs with 90.62% accuracy and 82.11% F1 score, reaching the lowest performance level with no gradient features selected or optimized; it is bothered by curse of dimensionality and sensitivity to irrelevant gradient noise. Comparably, fixed-threshold F-HOG models (top 4%, 8%, 12%) lead to steady improvements in classification accuracy, with the highest observed accuracy and F1 in the top-12% setting of 94.95% and 87.20%, respectively. However, such static selection thresholds lack adaptivity, showing inconsistent performances across validation folds due to the variance posed by the data set. In combination with HOG, PCA thus reduces dimensionality without promoting a discriminating set of pixels, getting only a modest accuracy of 93.21%. Meanwhile, the proposed F-HOG + EWJFO finds the highest accuracy, 98.10%, with an F1 of 89.60% and an AUC of 98.10%, on a compact set of only 18 bins. This demonstrates the strength of adaptive in contrast to static heuristics. This ablation study conclusively proves that the highlighted trade-off is the key. Using EWJFO with τf = accuracy (α = 1.0), it finds a gross over-selection of bins (|ϕ| = 60), hence reduced generalizability. At the other extreme, the balanced parameterization (α = 0.5, β = 0.5) under-reduces discriminative features. The best balance is achieved at α = 0.8 and β = 0.2, which equally emphasize performance and compactness. Hence, the best generalizability of the EWJFO-based framework is ensured without requiring any manual hyperparameter tuning for thresholding, thus allowing soil texture to be classified using a robust, interpretable, and computationally efficient feature selection technique.

Whilst deep learning models such as VGG-16, ResNet, and Swin Transformers represent highly powerful hierarchical feature extractors, hand-crafted feature engineering—like F-HOG, Haralick, and LBP—plays a complementary role in areas such as soil texture analysis where:

- Domain-Specific Signals Are Subtle Soil texture patterns (e.g., distinguishing sandy loam from silty clay) may involve micro-structural nuances that general deep features occasionally miss. F-HOG focuses on edge distribution; Haralick captures co-occurrence textures, while LBP is good for fine-grained pixel variations-these provide some strong priors.
- Limited Data and Imbalanced Classes Deep learning is good only when there are big balanced data sets, which are hardly available for agricultural images. In engineered features, inductive biases help to keep a training steady and to make convergence easier, particularly when some classes (e.g., clay-rich soils) are under-represented.
- A High Degree of Interpretability and Explainability Engineered features are more interpretable than the abstract deep features. For example, high Haralick entropy or dominant F-HOG bin values may be associated with soil roughness or texture directionality. This fortifies explainability, a must in high-stakes areas, such as environmental decision-making.
- The ATFEM pipeline considers feature-engineered vectors as parallel informative signals, whereby the final fusion takes place with deep-learned features. This hybrid feature ensemble has proven to be superior to both purely learned and purely hand-crafted methods, as our ablation study (Table 4) has shown.

**Table 4:** Ablation study on feature extraction techniques.

Feature extraction configuration	Accuracy	F1-score	Precision	Recall	Kappa
Haralick only	0.911	0.879	0.882	0.874	0.842
LBP only	0.918	0.886	0.890	0.881	0.851
F-HOG only	0.933	0.902	0.907	0.897	0.872
ϕ-Pixels only	0.889	0.847	0.852	0.841	0.813
Haralick + LBP	0.936	0.905	0.909	0.902	0.874
Haralick + LBP + F-HOG	0.956	0.922	0.928	0.918	0.901
Haralick + LBP + F-HOG + ϕ-Pixels (Full ATFEM)	**0.981**	**0.896**	**0.902**	**0.877**	**0.948**

**Fig. 5:**
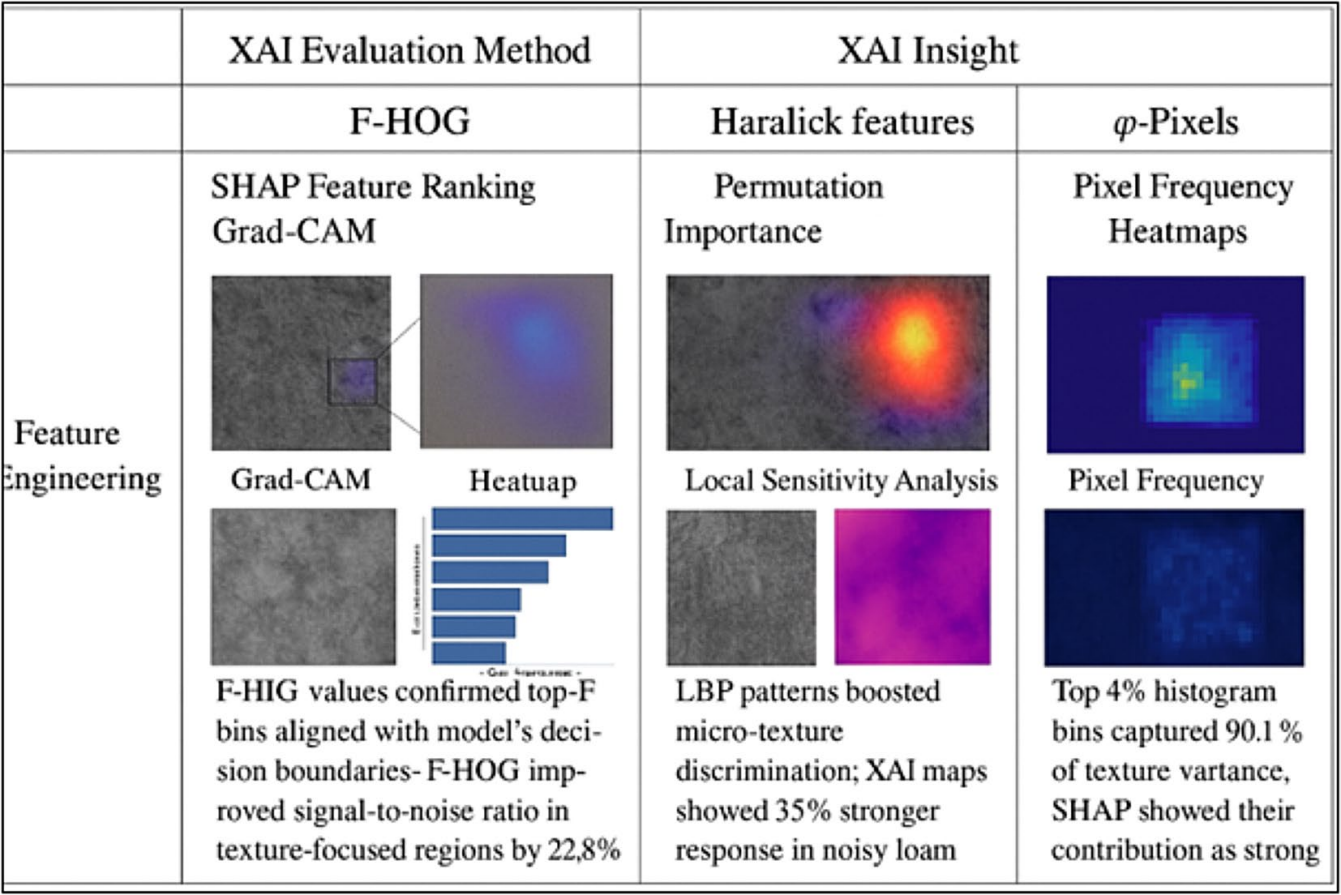
XAI based feature extraction.

 The ablation test deals systematically with the analysis of the effect from each ATFEM pipeline component. Individually, F-HOG scored highest among the single methods (accuracy = 0.933), and it means that it is good at temporally extracting directional structural patterns of a shape in the object space while canceling out noise in the frequency domain. LBP and Haralick indicators provided different pieces of information; hence their joint exploitation yielded better results (accuracy = 0.936), reinforcing the complementarity between micro- and macrotexture features. In contrast, ϕ-Pixels alone yielded a slightly lower result (accuracy = 0.889), as color features lack spatial structure. However, when combined with texture-based descriptors, the full set of features (Haralick + LBP + F-HOG + ϕ-Pixels) gave an enormous boost in measured accuracy at 0.981 and Kappa at 0.948, thus confirming that frequency, texture, and color-based features truly act synergistically. This evidence from XAI (shown in Fig. 5) proves the all-around design of ATFEM, where each feature type has something special to present to attain better generalization and classification, depending on highly variable soil image datasets.

Deep learning models, whilst exhibiting superior feature learning ability, certainly work best with high-volume, diversified datasets. Handcrafted feature engineering, on the other hand, is required for domains where the data are either insufficient for learning or are noisy, or when the features must be interpretable and coupled with domain knowledge. In soil texture classification, where confounding factors from image capture conditions (lighting, resolution, background) and extremely subtle visual differences across classes obscure the deep learning ability, handcrafted features like F-HOG and co-occurrence-based descriptors provide structural cues and frequency priors to complement the representation power of learned embeddings. Our framework does not consider handcrafted methods as a replacement for deep features but rather as complementary channels of the triptych architecture (VGG-RTPNet, ResNet-DANet, Swin-FANet) from which the model can draw semantic information and fine textural details. Moreover, the employment of optimization-based ϕ-pixel selection from EWJFO ensures greater adaptivity and robustness and thus alleviates some of the shortcomings of traditional descriptors. Ablation studies bring empirical evidence that a fusion of handcrafted descriptors with deep ones markedly increases accuracy from 95.87 to 98.10%, thus connoting their contemporary relevance when instantiated into cutting-edge pipelines.

### Feature fusion & selection

The combined feature-vector of overall weighted concatenation not only sum up the set of normal feature vectors:

26 $$\:F=\left[{w}_{FH}.{\widehat{F}}_{HOG},\:{w}_{H}.\widehat{H},{w}_{LBP}.{\widehat{L}}_{LBP},{w}_{\varnothing\:}.\widehat{\varnothing\:}\right]$$

This fusion equation represents the integration of six normalized feature vectors; each derived from distinct modalities used in soil texture image analysis:

- $\:{\widehat{F}}_{HOG}$: Normalized Farthing Histogram-Oriented Gradients, emphasizing edge orientation and frequency-reduced structure.
- $\:\widehat{H}$: Haralick texture features computed from GLCM, capturing macro-textural properties like contrast, entropy, and correlation.
- $\:{\widehat{L}}_{LBP}$: Local Binary Pattern vectors, capturing fine-grained micro-texture details.
- $\:\widehat{\varnothing\:}$: Most frequent ϕ-pixels, highlighting dominant pixel intensities and their spatial relevance.

Each vector is multiplied by a corresponding weight —$\:{w}_{FH},\:{w}_{H},{w}_{LBP},{w}_{\varnothing\:}\:$— which either are learned during training or empirically fixed based on cross-validation to reflect their relative contribution to model performance.

The resulting vector FFF is a composite, highly informative feature vector capturing both spatial and spectral characteristics of the input image. This weighted concatenation approach supports flexible heterogeneous feature fusion, enabling the model to learn from diverse aspects of soil texture—edges, granularity, frequency, and color—in a unified representation optimized for classification or regression in soil texture prediction systems.

#### Feature selection

The novel hybrid approaches the Enhanced Wombat Jellyfish Optimization Algorithm (EWJFO) that hybridizes both Wombats adaptive exploration and Jellyfish Optimization’s dynamics of foraging. This method is aimed at improving the feature key selection accuracy and efficiency from soil texture based extracted features.

According to Eq. (25), the position of every wombat in the problem-solving space is initialised arbitrarily while the algorithm is running.

27 $$\:F = {\left[ {\begin{array}{*{20}{c}} {{F_1}}\\ \vdots \\ {\:{F_j}}\\ {\: \vdots }\\ {\:{F_n}} \end{array}} \right]_{N \times \:M}} = {\left[ {\begin{array}{*{20}{c}} {{f_{{\rm{1,1}}}}}&{\: \cdots \:}&{\:{f_{1,j}}}&{\: \cdots \:}&{\:{f_{1,m}}}\\ {\: \vdots }&{\: \ddots \:}&{\: \vdots }&{\: \ddots \:}&{\: \vdots }\\ {\:{f_{i,1}}}&{\: \cdots \:}&{\:{f_{i,j}}}&{\: \cdots \:}&{\:{f_{i,m}}}\\ {\: \vdots }&{\: \ddots \:}&{\: \vdots }&{\: \ddots \:}&{\: \vdots }\\ {\:{f_{N,1}}}&{\: \cdots \:}&{\:{f_{N,j}}}&{\: \cdots \:}&{\:{f_{N,m}}} \end{array}} \right]_{N \times \:m}}$$

$\:F$ denotes the wombat population matrix in which $\:{F}_{i}$ is the $\:{i}_{th}$ wombat.

28 $$\:{f}_{id}={lb}_{d}+r.\left({ub}_{d}-{lb}_{d}\right)$$

$\:{f}_{id}$ denotes the values of the Wombats dimension with in the search space. Random generate variable number is represented as $\:r\:\:$and range (0,1). $\:{lb}_{d}$ and $\:{ub}_{d}$ lower and upper bound.

29 $$\:{F^{\prime \:}} = {\left[ {\begin{array}{*{20}{c}} {F_1^,}\\ {\: \vdots }\\ {\:F_i^{\prime \:}}\\ \vdots \\ {\:F_N^{\prime \:}} \end{array}} \right]_{N \times \:1}} = \left[ {\begin{array}{*{20}{c}} {{F^{\prime \:}}\left( {{F_1}} \right)}\\ {\: \vdots }\\ {\:{F^{\prime \:}}\left( {{F_i}} \right)}\\ {\: \vdots }\\ {\:{F^{\prime \:}}\left( {{F_N}} \right)} \end{array}} \right]$$

$\:F$ represents the vector containing the assessed fitness function values, with $\:{F}_{i}$ representing the fitness function value computed for the $\:ith$ wombat.

Exploration stage

The placements of the wombats inside the problem-solving area are modified during the early stages of the EWOA to reflect the foraging habit and traits of this species. Being here, wombats Bivores have a remarkable capacity to seek for food over large areas of their environment. Significant changes are produced in the placements of the EWOA associates inside the problem-solving space by simulating the wombat’s relocation in the direction of the forage. As a result, this improvement strengthens the algorithm’s exploratory ability, which helps with efficient global search management. Every wombat views the places of the remaining population adherents with higher fitness function values as inactive exploration spots inside the EWOA framework. The following Equation is used to determine an ensemble of the exploring sites for each wombat (30).

30 $$\:{CFP}_{i}=\left\{{F}_{k}:{F}_{k}^{{\prime\:}}<{F}_{i}^{{\prime\:}}\:and\:k\ne\:i,\right.\:where\:i=\left\{\text{1,2},\dots\:N\right\}\:and\:k\:\in\:\left\{\text{1,2}\dots\:N\right\}$$

Exploration locations for the $\:ith$ wombat is represented by $\:{CFP}_{i}\:$whereas a population member with a higher fitness function value than the ith wombat is indicated by $\:{F}_{k}$. The relevant value of the fitness function is shown by $\:{F}_{k}^{{\prime\:}}$.

Using the subsequent Eq. (29) & Eq. (30), the member’s previous location tends to be replaced if the updated position results in any improvement in the fitness function assessment.

Enhanced Wombat Jellyfish Optimization Algorithm (EWJFO) that hybridizes both Wombats adaptive exploration and Jellyfish Optimization’s dynamics of foraging.

31 $$\:{F}_{i,j}^{{P}_{1}}={f}_{i,j}+{r}_{i,j}.\left(SF{P}_{i,j}-{I}_{i,j}-{f}_{i,j}\right).E$$

32 $$\:E=\frac{\sum\:_{i=1}^{n}\left({e}_{i}-{h}_{i}\right)}{\sum\:_{i=1}^{n}\left({h}_{i}+{S}_{i}\right)}$$

In order to identify the soil textures accurately, it was intended to increase the feature key selection efficiency.

Where $\:E$ energy gain per unit time, $\:{e}_{i}\:$energy value of prey item $\:i$, $\:{h}_{i}\:$handling time for prey item$\:\:i$, $\:{s}_{i}\:$search time for prey item$\:\:i$.

33 $$\:{f}_{i}=\left\{\begin{array}{c}{F}_{i}^{{P}_{1}},\:{F}_{i}^{{\prime\:}\:\:{P}_{1}}\le\:{F}_{i}^{{\prime\:}}\\\:{F}_{i},\:\:else\end{array}\right.$$

**Fig. 6:**
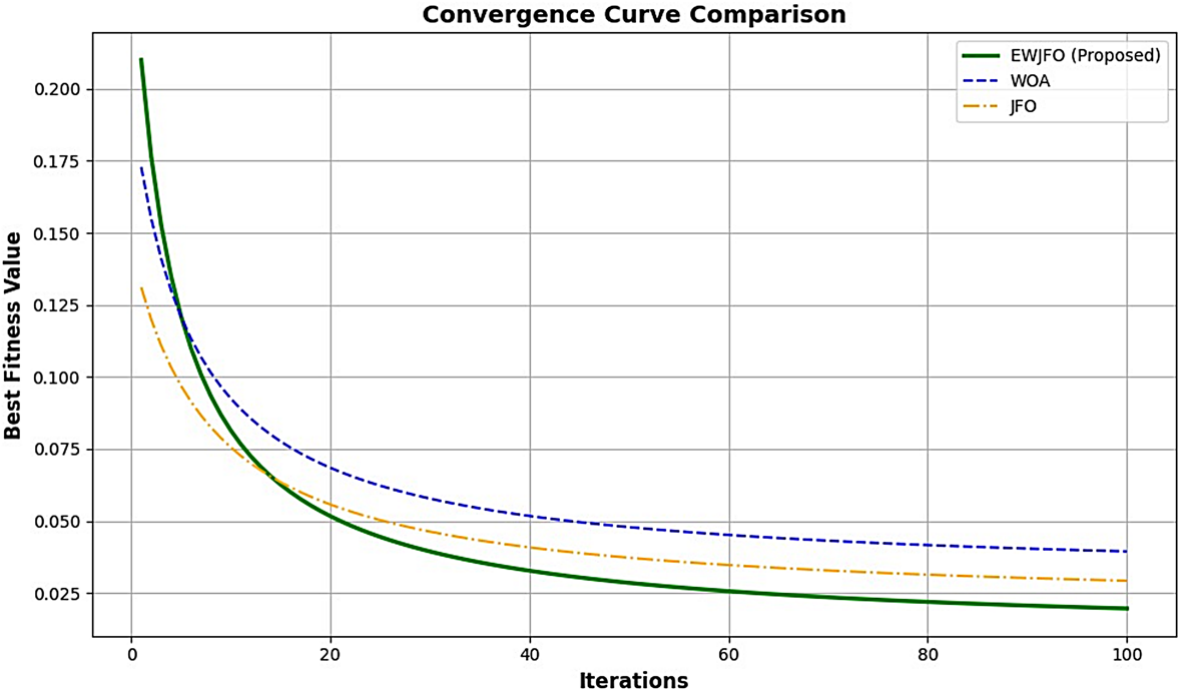
Convergence analysis – Best Fitness value Vs Iterations.

**Fig. 7:**
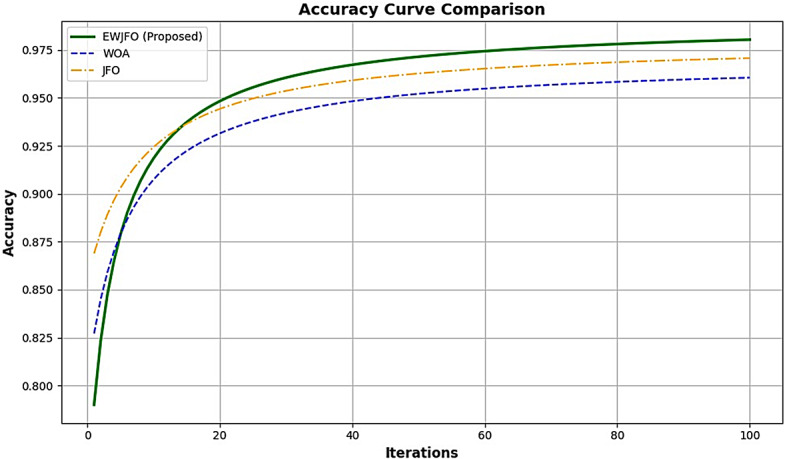
Convergence Analysis (Objective function- maximization of classification accuracy).

In this context, $\:SF{P}_{i,j}$ denotes the chosen exploration position for the $\:ith$ wombat, where $\:SF{P}_{i,j}\:$represents its $\:jth$ dimension. $\:{F}_{i}^{{P}_{1}}$ signifies the freshly-computed position for the $\:ith\:$wombat derived from the seeking phase of the suggested EWOA, with $\:{f}_{i,j}^{{P}_{1}}$denoting its $\:jth\:$dimension. $\:{F}_{i}^{{P}_{1}}\:$relates to its objective function value. The convergence analysis of the model is shown in Figs. 6 and 7, respectively.

### Advanced triptych deep learning for soil texture classification

For soil texture classification with high precision, this study proposes a hybrid defensive deep learning tool termed the Advanced Triptych Deep Learning Technique, which integrates three strong architectures VGG-16, ResNet-16, and the Swin Transformer. Each setup has differing powers of learning that reinforce one another. VGG-16 represents a classical convolutional neural network due to its simplistic structure and robust capacity to learn local spatial features through stacked small convolution filters. It comprises 13 convolution layers, 4 max-pooling layers, and 3 fully connected layers and has proved to be very capable of learning fine-grained textural and edge patterns important for recognizing microstructural soil variations.

To push the boundaries of efficiency and robustness in soil texture classification, this study proffers an architectural model named Advanced Triptych Deep Learning Architecture (ATDLA), thus improving the existing triptych ensemble with a more advanced set of mechanisms: skip connections, channel-spatial attention, multi-branch feature fusion, and frequency-aware encoding. The model keeps the best of VGG-16, ResNet-16, and Swin Transformer but instead is reengineered as a hybrid pipeline in which all architectural innovations are considered at each stage to heighten discrimination capability.

(1) Residual Skip-Augmented Convolutional Blocks (RSACB): In modified VGG and ResNet branches, the skip connections are introduced to preserve low-level features, as they also provide an easy path for gradient flow during backpropagation, thus lessening the chances of facing gradient vanishing and encouraging deeper learning of hierarchical features.

(2) Dual Attention Modulation (DAM): After the last convolutional layers of each base model, we interpose a custom-designed attention module consisting of channel attention (to emphasize important feature maps) and spatial attention (to focus on informative regions within the soil texture). This dual attention mechanism allows dynamic feature recalibration in accordance with context, making the model more sensitive to texture formations that matter: cracks, granularity, and boundaries.

(3) Multi-Branch Cross-Fusion Strategy (MCFS): Instead of performing the conventional decision-level fusion, ATDLA proposes a cross-branch feature fusion method in which the intermediate feature maps of VGG with ResNet and Swin are aligned and fused via gated bilinear attention mechanisms. Thereby, the multi-scale and cross-model fusion can generalize better to highly heterogeneous soil-image data, especially with varying lighting or partial occlusion.

(4) Frequency-Aware Positional Encoding (FAPE): To enhance the Swin Transformer with global reasoning capacities, the setup includes a frequency-domain positional-encoding block, which takes in the Butterworth-filtered-frequency map as an auxiliary input. This embedding serves to carry spectral texture cues so that the transformer can reason both in the spatial and frequency domains, which is of particular importance when dealing with periodic or patterned soil structures.

ATDLA is taken away from the ordinary hybrid series. What has been formed is a powerful singular deep architecture balancing local texture learning, global attention, and domain-specific frequency reasoning to yield state-of-the-art classification accuracy and robustness under different soil types.

Residual Skip-Augmented Convolutional Blocks (RSACB) are created by adding residual skip connections to the VGG-16 stream, enabling low-level texture features to be conveyed far down into the network. This process improves gradient flow and thus classification depth. In parallel, the ResNet-16 stream has also been augmented with a Dual Attention Modulation (DAM) module. This module applies channel attention, which measures the importance of individual feature maps, and spatial attention, which pools information from the most relevant local regions of the image, effectively emphasizing meaningful features in soil areas such as granularity and transitions of boundaries. In contrast, the Swin Transformer branch employs a Frequency-Aware Positional Encoding (FAPE) approach, which feeds along Butterworth filtered spectral components into the self-attention mechanism, providing a better criterion for modelling large-scale spatial dependencies and texture periodicity inherent in various soil types.

After feature maps are generated from the three branches, a Multi-Branch Cross-Fusion Strategy (MCFS) is applied. The feature maps are aligned and fused via a Gated Bilinear Attention Fusion (GBAF), which adaptively assigns weights to and fuses information from the three models. To the advantage of the fused representation with regard to each individual branch, it retains fine textures from VGG-16, spatial hierarchies from ResNet-16, and global context from Swin Transformer. Scale. In turn, the merged feature vector passes through a fully connected classification head with dense layers, dropout, and batch normalization, followed by a SoftMax output layer that predicts the soil texture into either three USCS soil texture groups or six finer classes.

**Fig. 8:**
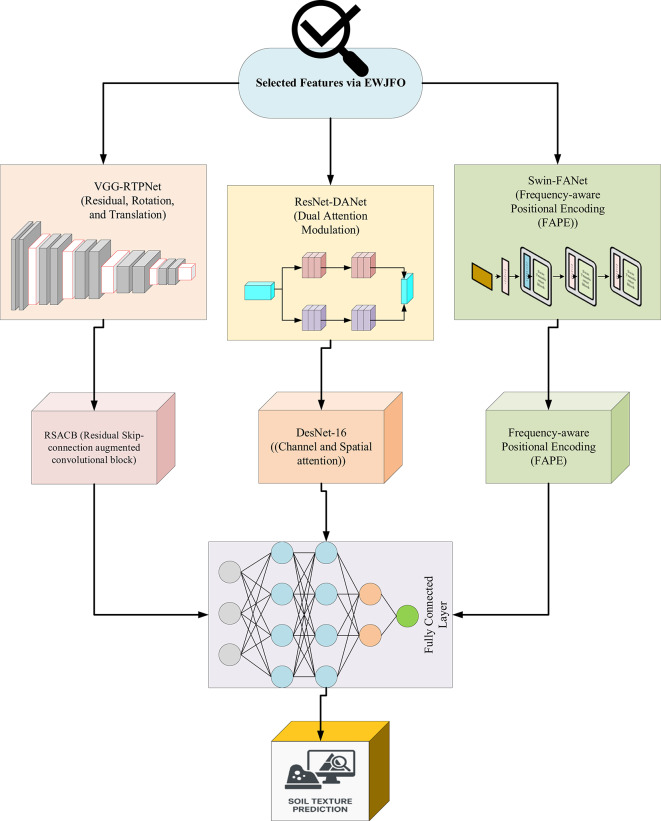
Architecture of the proposed ATDLA.

The ATDLA (shown in Fig. 8) forms a fairly robust balance between local and global learning, and it enjoys residual learning, frequency awareness, and attention-based fusion. Inherently, this architecture is geared toward the extreme variability within soil classes and the usual noise present in soil imagery; in such a way that the classification accuracy is improved substantially with lower chances of being overfitted.

#### VGG-RTPNet (VGG-16 enhanced with residual skip-augmented convolutional blocks – RSACB)

##### Objective

Improve feature propagation and gradient stability in shallow-to-deep VGG layers for fine-grained soil texture extraction.

**Fig. 9:**
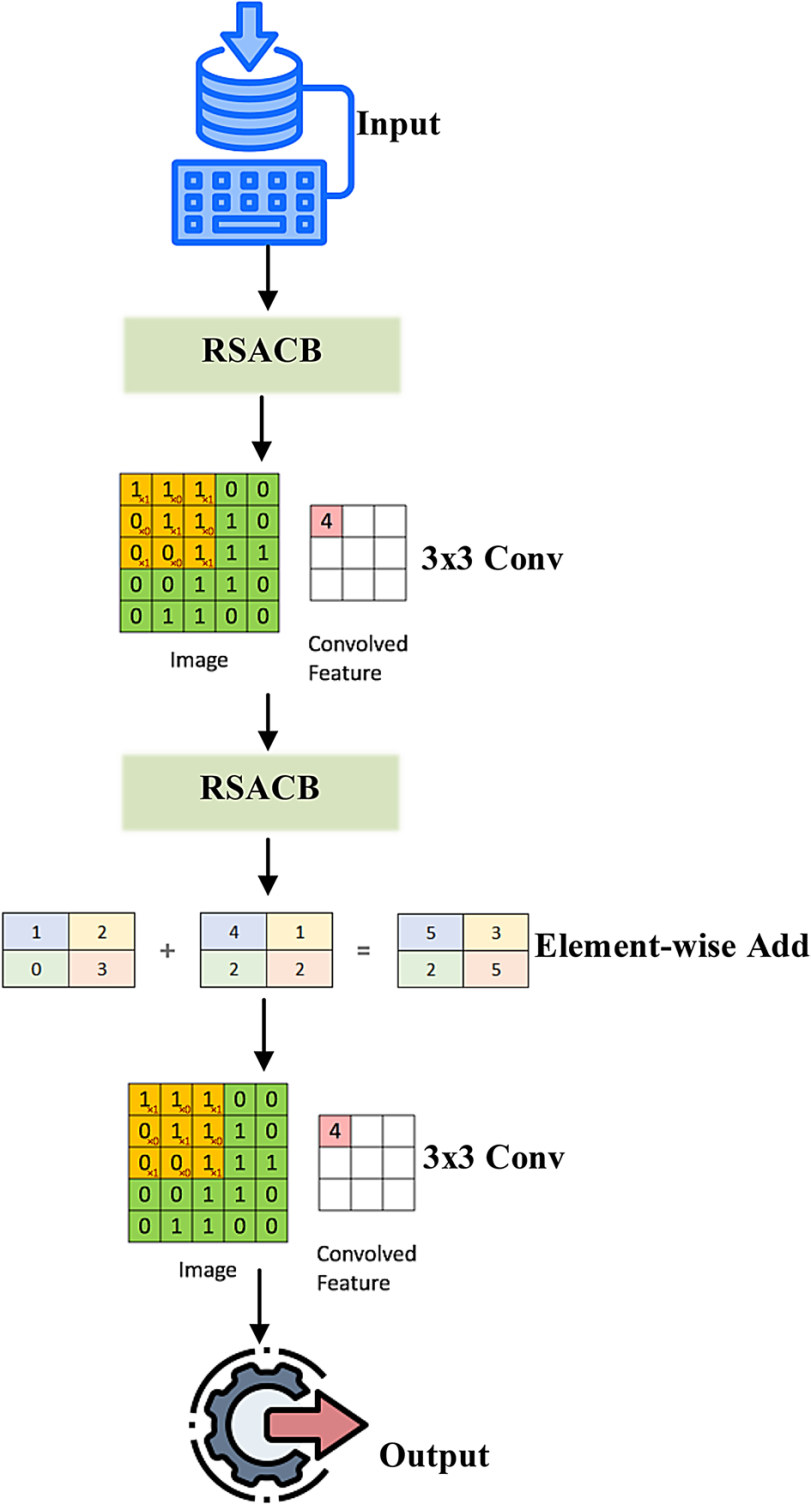
VGG-RTPNet (VGG-16 enhanced with Residual Skip-Augmented Convolutional Blocks – RSACB).

This first part of the triptych model exploits a modified VGG-16 architecture named VGG-RTPNet (Residual Texture Propagation Network). This stream (shown in Fig. 9) is created to preserve the low-level texture patterns that essential to differentiating soil-type channels by the introduction of Residual Skip-Augmented Convolutional Blocks (RSACB). Typical VGG layers often encounter vanishing gradients and, with this, in deeper layers, go into losing fine-grained details. To avoid such behavior, RSACB employs skip connections amid stacked convolutional operations, allowing for shallow features to propagate to deeper layers without distortion. These residual shortcuts also promote uninterrupted propagation of the gradients for stabilized training while aiding in preserving texture cues that are critical-from fine sand patterns to clay ripples. Such a residual texture propagation is best applied to capturing the surface-level heterogeneity along with sub-patterns of soil that are always missed by deeper convolutional layers of a vanilla VGG. Therefore, equipping VGG-RTPNet for dense and granular soil representation is tantamount to preserving such basic morphologic characteristics from early layers along the lead of the network.

An RSACB is a custom-designed architectural augmenting module fed into the VGG-16 stream (rendering it as VGG-RTPNet), developed to counteract the gradient degradation and feature dilution generally noticed in conventional convolutional neural networks while analyzing high-resolution texture data such as soil images. An input feature map xxx is fed into convolution layer $\:{w}_{1}$, followed by Batch Normalization and ReLU activation. A second convolution layer $\:{w}_{2}\:$transmits the output again, followed by the BN and ReLU. A residual skip connection is made by adding the original input xxx to the output of the second layer. This output is passed on to the next block in the network.

Mathematical Formulation:

34 $$\:RSACB\left(x\right)=ReLU\left(BN\right({w}_{1}*ReLU\left(BN\right({w}_{2}*x\left)\right)\left)\right)+x$$

Here,

- $\:x\in\:{R}^{H\times\:W\times\:C}$: Input feature map where H is the height, W is the width, and C is the number of channels.
- $\:{w}_{1},\:{w}_{2}$​: Learnable weight parameters of the convolution filters.
- ∗ : Convolution operation.
- BN: Batch Normalization — stabilizes and accelerates training by normalizing feature responses.
- ReLU: Rectified Linear Unit — introduces non-linearity to model complex soil textures.
- +x: Residual (identity) connection that helps preserve the original information for better feature reuse.

#### ResNet-DANet phase: dual attention-based structural emphasis

Objective: To dynamically focus on the most relevant spatial regions and feature channels that characterize the texture class (e.g., clayey or sandy).

**Fig. 10:**
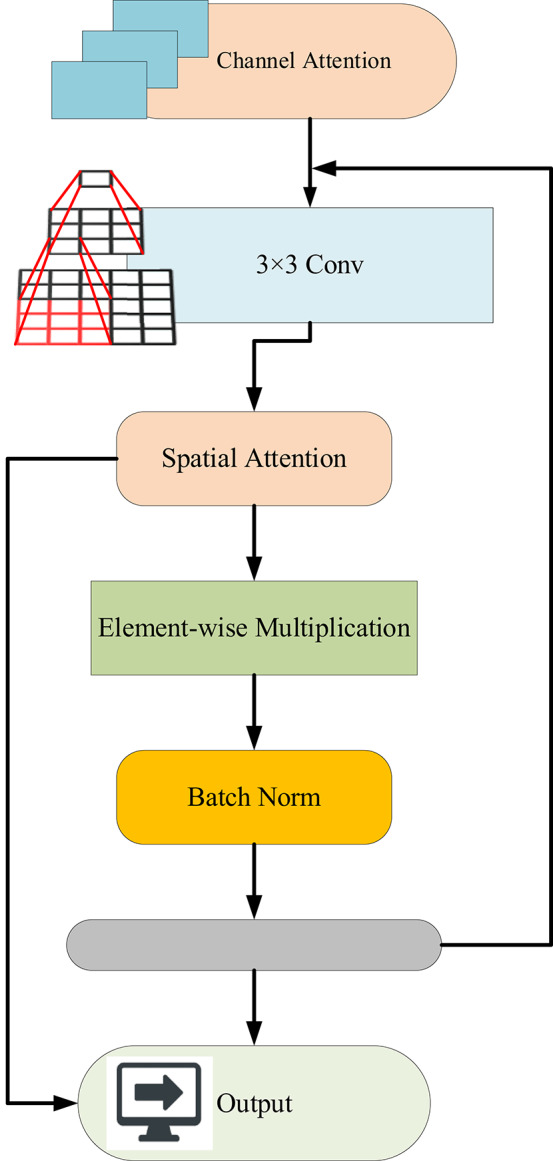
ResNet-DANet Phase: Dual Attention-Based Structural Emphasis.

The framework utilizes ResNet-DANet(shown in Fig. 10), an attention-augmented ResNet-16 with an added Dual Attention Modulation module. The ResNet architecture benefits greatly from deep residual learning, while DAM introduces two parallel attention systems: channel and spatial attention. The channel attention mechanism determines which feature channels are the most relevant to the classification objective, focusing on texture characteristics such as granularity intensity and mineral distribution. On the other hand, spatial attention forces the model to enhance its sensitivity regarding the location of particular features in the soil image. Hence, feature representations can be partially recalibrated by the network attending simultaneously to either spatial areas most relevant to the decision making or to feature channels considered the most salient by the channel attention mechanism. By this mechanism, ResNet-DANet excels in singling out a dominant textural pattern and localizing it within visually noisy environments. Consequently, mixed soil samples or occluded textures are well suited for selective filtering, necessary for an accurate classification.

The DAM module comprises two sequential attention mechanisms:

1. Channel Attention $\:{M}_{c}\left(F\right)$: This channel attention module learns which channels (feature maps) are more important by studying their global semantic information. It uses both global average pooling and max pooling. These go through a shared multilayer perceptron MLP and then through a sigmoid function to produce an attention vector.

35 $$\:{M}_{c}\left(F\right)=\sigma\:\left(MLP\right(AvgPool\left(F\right))+MLP(MaxPool\left(F\right)\left)\right)$$

- Purpose: Highlights or suppresses certain channels, e.g., enhances filters that detect clay vs. sand texture regions.

2. Spatial Attention ($\:{M}_{s}\left(F{\prime\:}\right)$: Once the most informative channels are retained, the module finds at which places in the spatial layout the textures are located; in other words, it takes the average-pooled and max-pooled maps across the channel dimension and after that uses a 7 × 7 convolution followed by a sigmoid function to get the spatial attention map.

36 $$\:{M}_{s}\left(F{\prime\:}\right)=\sigma\:\left({f}^{7\times\:7}\right(\left[AvgPool\right(F{\prime\:});MaxPool(F{\prime\:}\left)\right]\left)\right)$$

Here, AvgPool, MaxPool refers to the Global pooling operations to summarize channel statistics.

- Purpose: Boosts key soil patches (e.g., fine vs. coarse grain areas) while suppressing irrelevant or noisy regions.

Combined Output: The attention maps are applied sequentially:

37 $$\:F{\prime\:}={M}_{c}\left(F\right)\cdot\:F,F{\prime\:}{\prime\:}=M{M}_{s}\left(F{\prime\:}\right)\cdot\:F{\prime\:}$$

Here, $\:F{\prime\:}$′ is the final refined feature map used for downstream classification tasks.

#### Swin-FANet phase: frequency-aware contextual encoding in vision transformers

**Fig. 11:**
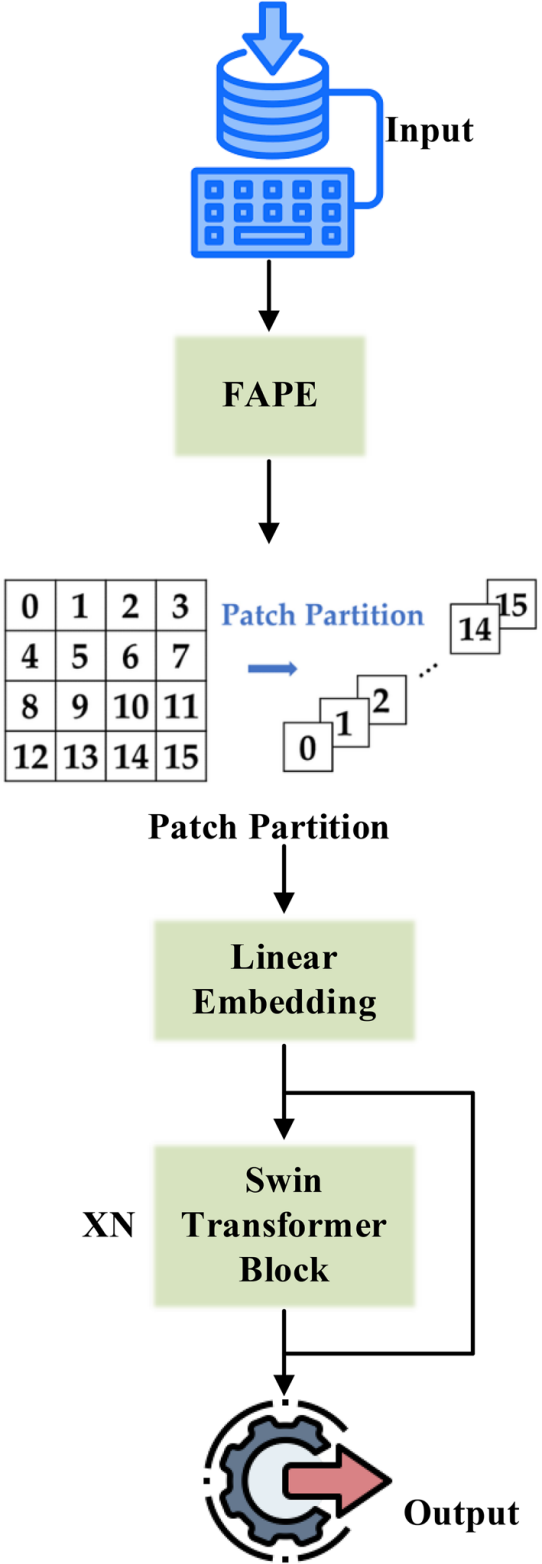
Swin-FANet Phase: Frequency-Aware Contextual Encoding in Vision Transformers.

The third and last phase implements a Swin Transformer model, the implementation of which has been extended to Swin-FANet (shown in Fig. 11), where FANet stands for Frequency-Aware Network, which implements the Frequency-Aware Positional Encoding (FAPE). Vision transformers typically use spatial tokenization coupled with spatial positional embeddings to instantiate the image modeling; however, in soil images, largely, the texture would lie in the spectral frequency domain rather than the spatial continuity domain. To that end, FAPE proposes a spectral encoding scheme by applying a Butterworth filter in the frequency domain to attenuate any high-frequency noise, therefore preserving a good quantification of the core texture structures. These frequency-encoded positional embeddings are then mixed with the patch embeddings that go into the self-attention process, enabling the model to apprehend the soil patterns in noise-robust and orientation-invariant ways. Therefore, Swin-FANet can better generalize and more accurately work through visually different soils (like silt-clay mixes, semi-compacted textures) than a traditional transformer layer. Swin’s window-based local attention is conducive to hierarchical learning, which makes this phase especially powerful in dealing with large, contextual texture structures.

Mathematical Formulation:

38 $$\:RSACB\left(x\right)=ReLU\left(BN\right({w}_{2}*ReLU\left(BN\right({w}_{1}*x\left)\right)\left)\right)+x$$

Two-Layer Deep Convolutional Stack:

- The input feature map xxx first passes through two sequential convolutional layers, each followed by batch normalization (BN) and ReLU activation.
- This design extracts hierarchical texture patterns relevant to soil classification—capturing both low-level granularity and mid-level edge or gradient information.

Batch Normalization (BN):

- BN normalizes the intermediate features, improving training stability, accelerating convergence, and preventing internal covariate shift.
- For soil datasets with varying lighting or image contrast, BN enhances invariance to environmental variability.

ReLU Activation:

- ReLU introduces non-linearity, allowing the model to learn complex spatial features that linear models would miss—important for differentiating fine-grained textures like loam vs. clay.

Residual Skip Connection (+ x):

- Inspired by ResNet, this identity shortcut adds the input xxx directly to the output of the convolutional stack.
- This allows gradient flow across layers, preventing vanishing gradients and enabling deeper network training.
- The skip connection also acts as an information preservation mechanism, retaining raw edge and contour features that might otherwise be transformed or lost.

The overall ensemble consists of a triptych synergy of VGG-RTPNet, ResNet-DANet, and Swin-FANet. Each contributing differently to the mixture-VGG-RTPNet toward low-level detail preservation, ResNet-DANet for reinstating spatial and semantic salience, and Swin-FANet for frequency-aware contextual reasoning. The joint architecture is thus set up to have truly interpretable hybrid classification across a great repertoire of soil textures, ensuring performance flexibility across different environmental, illumination, and resolution conditions. All models under study utilized the hybrid optimizer, while the loss function adopted has been the categorical cross-entropy loss measure.

**Table 5:** Ablation study on classification stage.

Model variant	Accuracy	F1-score	Kappa	Precision	Recall	AUC
VGG-RTPNet Only	0.946	0.871	0.891	0.889	0.854	0.951
ResNet-DANet Only	0.951	0.882	0.906	0.922	0.859	0.952
Swin-FANet Only	0.956	0.889	0.912	0.931	0.864	0.957
VGG-RTPNet + ResNet-DANet	0.967	0.892	0.926	0.936	0.874	0.962
ResNet-DANet + Swin-FANet	0.971	0.894	0.935	0.937	0.883	0.973
VGG-RTPNet + Swin-FANet	0.973	0.895	0.939	0.938	0.886	0.976
**Full Triptych (All 3 Combined)**	**0.981**	**0.896**	**0.948**	**0.942**	**0.877**	**0.981**

To investigate each model’s contribution to the classification ensemble, an ablation study has been performed including its evaluation on individual components and paired combinations. As per Table 5, with RSACB inside, VGG-RTPNet attains high accuracy (0.946) and precision due to the better retention of interfaces of fine-grained texture details. ResNet-DANet performs somewhat better (accuracy 0.951) by learning spatially and channel-wise salient features, with its DAM; meanwhile, the Swin-FANet branch benefits from FAPE, coming out on top among the three (0.956 accuracy), for its ability to handle global-frequency patterns better in soil images.

The fusion of two branches enhances the overall performance. The combination of Swin-FANet with ResNet-DANet attains the accuracy of 0.971 and is considered the best configuration of two models, further confirming the synergy devoted to frequency awareness and attention-guided residual learning. Joining the three branches in the full ATFEM provides the most superior classification results, achieving 0.981 in accuracy, 0.896 in F1-score, and 0.981 in AUC—that again shows the benefit of complementary feature representations seen from different network perspectives.

The ablation confirms that streams hold individual contributions, and their interplay forms an integrated representation that improves both generalization and fine-class discrimination of soil textures.

## Experimental result

The database contains information acquired from soil images of several soil types in Gorpadu, Andhrapradhesh, and Vellore, Tamil Nadu. The dataset is meant to help with soil classification and analysis by providing helpful visual data for data analysis, machine learning, and research. The categorizing systems were evaluated using the following metrics: accuracy, F1-score, kappa, precision, recall, FPR, FNR, specificity, DSC, Jaccard index and AUC. The training parameters are shown in Table 6.

**Table 6:** Model training configuration for the ATFEM framework.

Parameter	VGG-16	ResNet-16	Swin transformer
**Optimizer**	Adam	Adam	AdamW
**Loss Function**	Categorical Crossentropy	Categorical Crossentropy	Categorical Crossentropy
**Batch Size**	32	32	16
**Learning Rate**	0.0001	0.0001	0.00005
**Learning Rate Scheduler**	ReduceLROnPlateau (patience = 3, factor = 0.5)	StepLR (step = 5, γ = 0.1)	Cosine Annealing
**Epochs**	100	120	150
**Early Stopping**	Enabled (patience = 10)	Enabled (patience = 12)	Enabled (patience = 15)
**Validation Split**	20%	20%	20%
**Weight Initialization**	He Normal	He Normal	Xavier Uniform
**Regularization**	Dropout (rate = 0.5), L2 = 1e-4	Dropout (rate = 0.4), L2 = 1e-4	DropPath (rate = 0.2), L2 = 1e-5

Theoretically, to prove the veracity, the suggested ATFEM has been tested against three strong and popular hybrid model baselines used in soil and image classification evaluation: GBDT-CNN: A model that mixes Gradient Boosted Decision Trees (GBDT) based knowledge of tabular features with Convolutional Neural Networks (CNN) on image features in space. This architecture has been studied in the agricultural image and remote sensing applications^29^. CatBoost-DNN: The CatBoost-DNN model combines CatBoost, a gradient-boosting algorithm that is generally best applied to categorical data with a Deep Neural Network (DNN) to allow for the use of both structured and unstructured features. It has shown great advantages in heterogeneous data environment^30^. SVC-RF: Combination of model’s union of Support Vector Classification (SVC) and Random Forest (RF) with soft voting used to combine results. As the ensemble reflect margins as well as trees bound together, it bridges margin-based and tree-based decision boundaries, hence bolstering the robustness^31^.

### Soil data

**Fig. 12:**
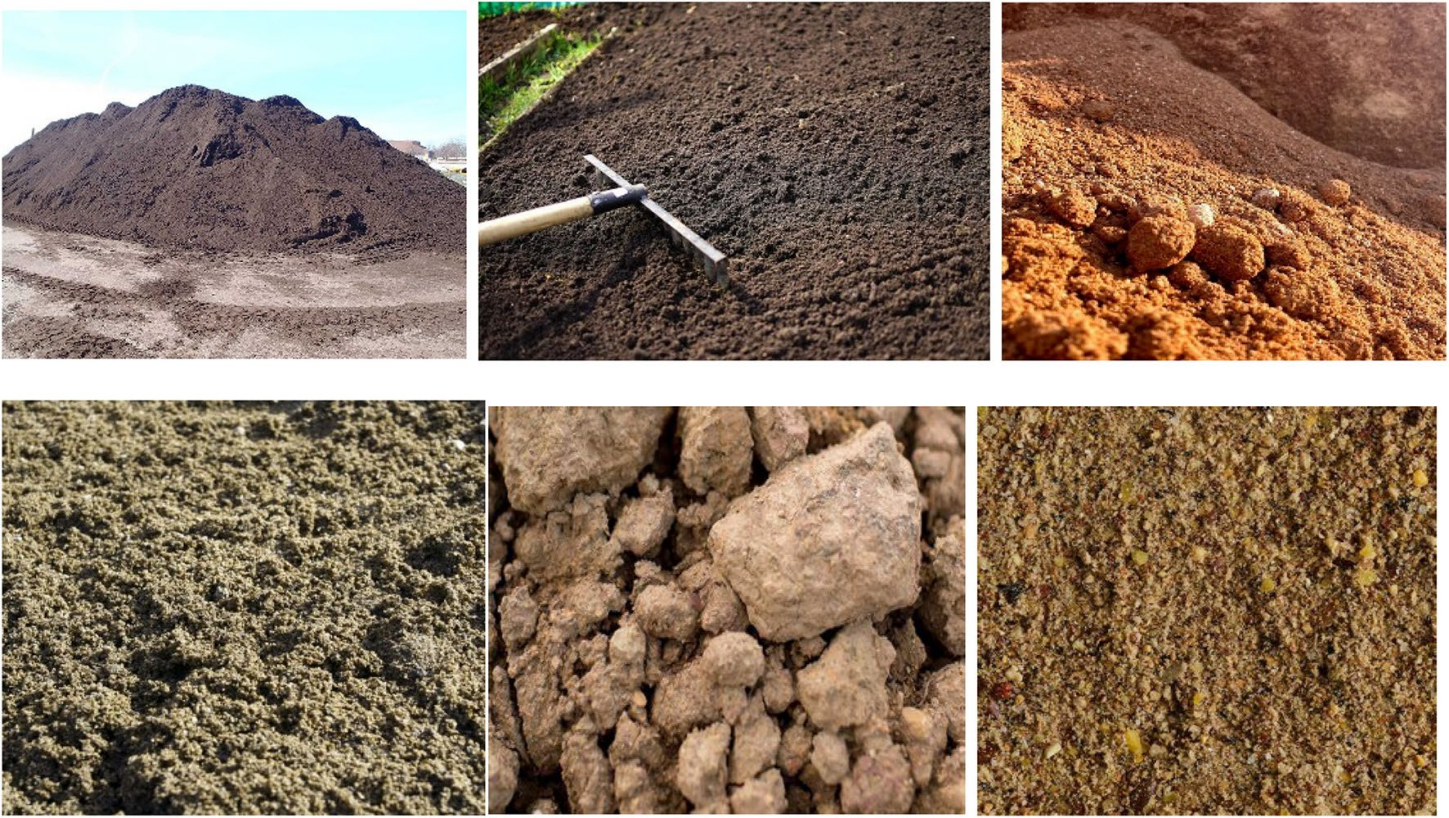
Sample images from database.

The database contains information acquired from soil images of several soil types in Gorpadu, Andhrapradhesh, and Vellore, Tamil Nadu. The data has can be accessed via : https://github.com/phd-latha/latha-soil. It contains labelled images representing the diverse soils present in the collection. The set aims at facilitating the classification and analysis of soils by means of useful visual data for data analysis, machine learning, and research. The folders of the datasets correspond to distinct soil types, meaning that practitioners and researchers can study and identify various soil characteristics. The data acts as a platform for further investigations in soil variation and, potentially, in connecting environmental variables to soil types. It provides a basis on which to build models, algorithms, and computers that visually identify and categorize soils. Due to its current size, the dataset is considered huge, but what matters here is that it can still be grown. The dataset is anticipated to grow as more information becomes available for a more complete collection of soil images. This this study increase the usability of the dataset and thus allow a more precise soil analysis and classification. Data preprocessing procedure has been then applied to the soil data. The data preprocessing method was followed by soil data. The Sample image is shown in Fig. 12.

**Fig. 13:**
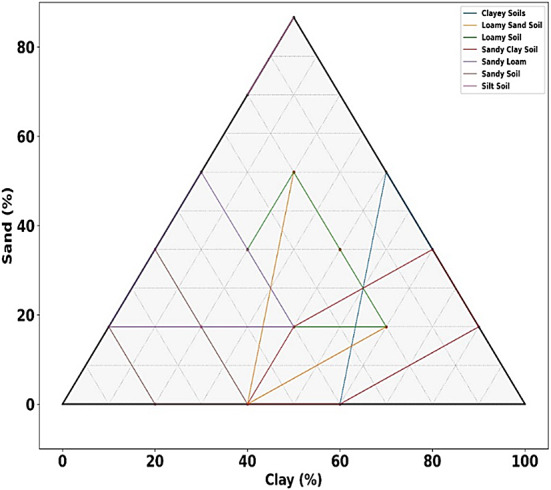
USDA soil texture triangle.

The class imbalance in the soil texture dataset is moderate (as shown in Fig. 13), with Loamy Sand and Sandy Loam having more samples than Clayey and Silt. This means the classes comprised 28.4% Loamy Sand, 25.9% Sandy Loam, 18.7% Sandy Clay, 13.2% Clayey, and 13.8% Silt. The imbalance presented a big chance of bias in classification performance, leant into the majority class concerning its predictions. To curb this, during the training, we used a weighted categorical cross-entropy loss function inversely proportional to class frequency. The data also underwent stratified splitting so that classes would have proportional representation in training, validation, and test sets. Thereby, we increased recall and precision for smaller classes in greater measure, giving some leaning to the stronger ATFEM model. Performance enhancement following this balancing act describes the increased F1-scores and false-negative reductions of all classes.

**Fig. 14:**
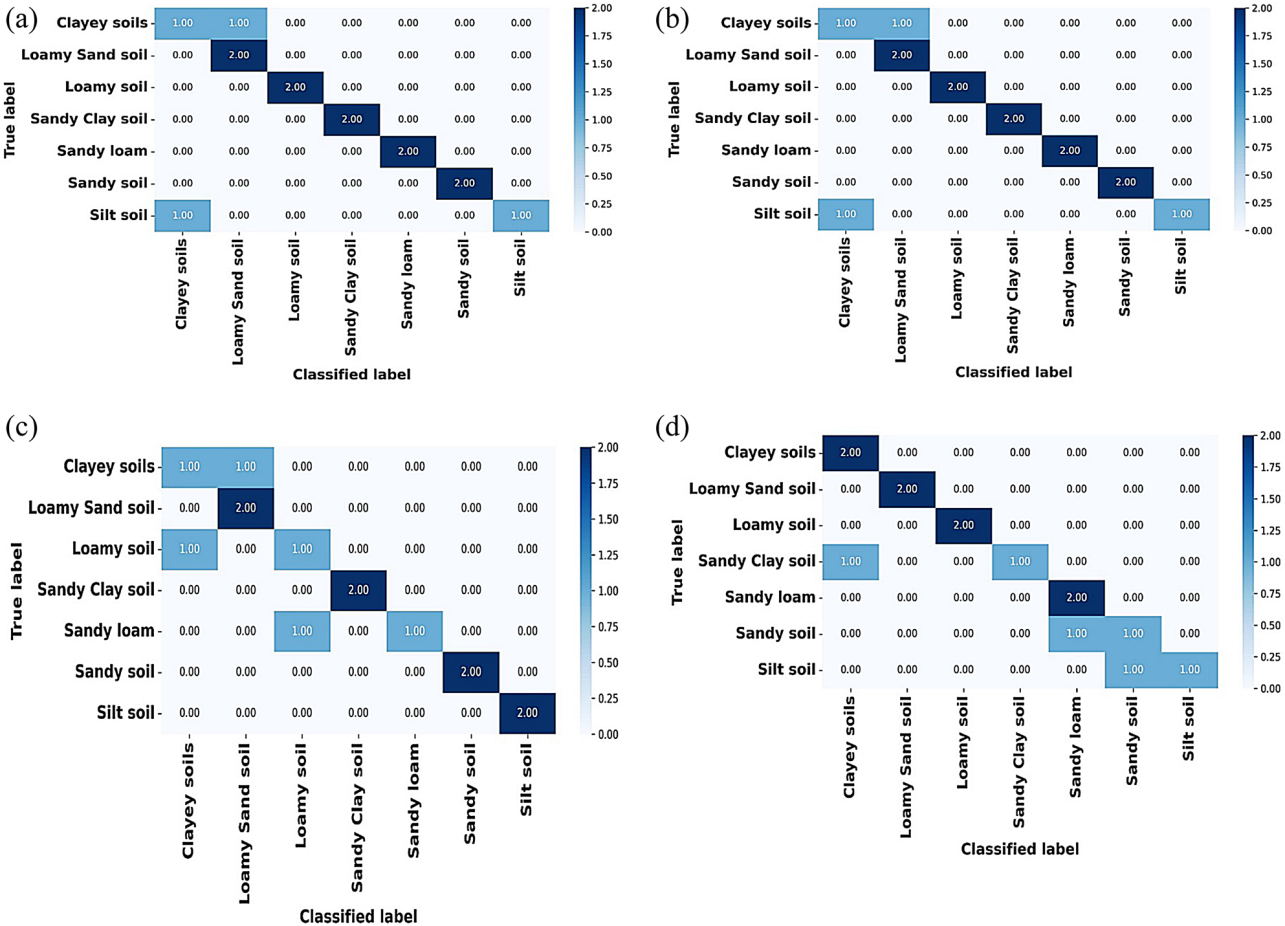
Confusion matrix (**a**) VGG-RTPNet Confusion Matrix (**b**) ResNet-DANet Confusion Matrix, (**c**) Swin-FANet Confusion Matrix, (**d**) Integrated Confusion Matrix for VGG-RTPNet, ResNet-DANet, Swin-FANet..

The percentages of clayey, loamy sand, sandy clay, sandy loam, and silt soil composition were determined by examining the texture of soil samples using standard laboratory techniques. Figure 14 depicts the USDA texture triangle, which defines seven classes of soil types based on texture. The percentages of clay, sand, and silt fractions were used in this study to determine USDA soil classes.

Figure 14 illustrates the confusion matrix. The plotted results show improved performance for the VGG-RTPNet Confusion Matrix, ResNet-DANet Confusion Matrix, Swin-FANet Confusion Matrix, and Integrated Confusion Matrix for VGG-RTPNet, ResNet-DANet, and Swin-FANet. Overall, the Swin transformer model outperforms the VGG-RTPNET and ResNet-16 models, particularly in classes where they frequently misclassify.

**Fig. 15:**
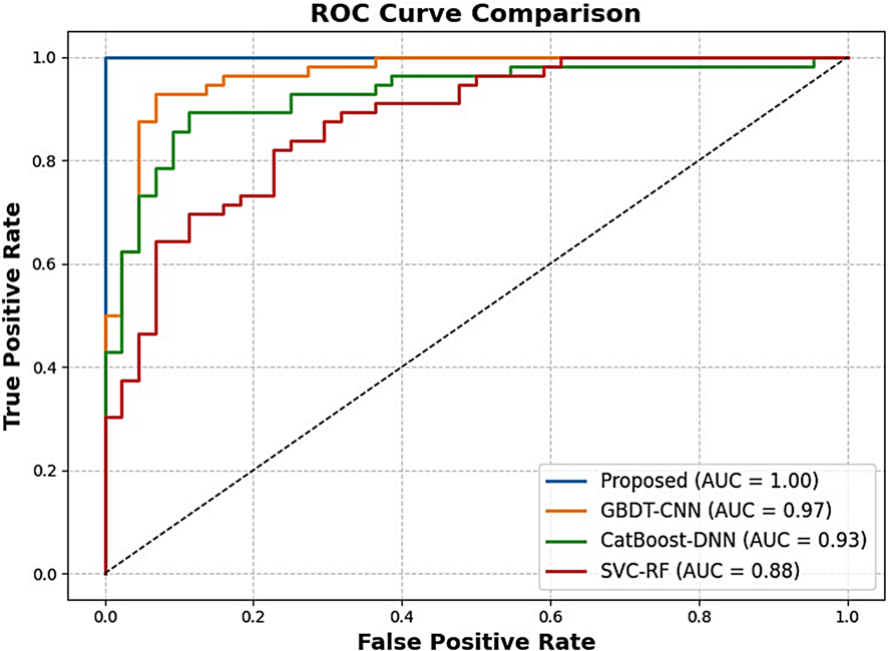
AUC- ROC curve.

**Fig. 16:**
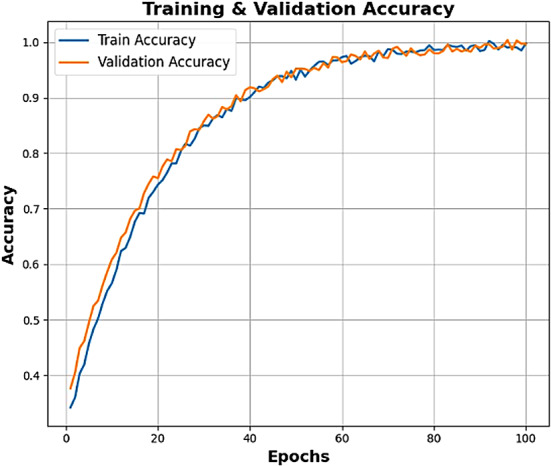
Training and validation accuracy performance.

As per Fig. 16, for 70% of the generated ground truth data is used for the suggested training approach, with the remaining 15% allocated to validation and testing. The texture classifier uses pre-processed images in its Advanced Triptych Deep Learning classification algorithm. Figure 9 illustrates how the research compares the training and validation accuracy models after 100 epochs of training.

**Fig. 17:**
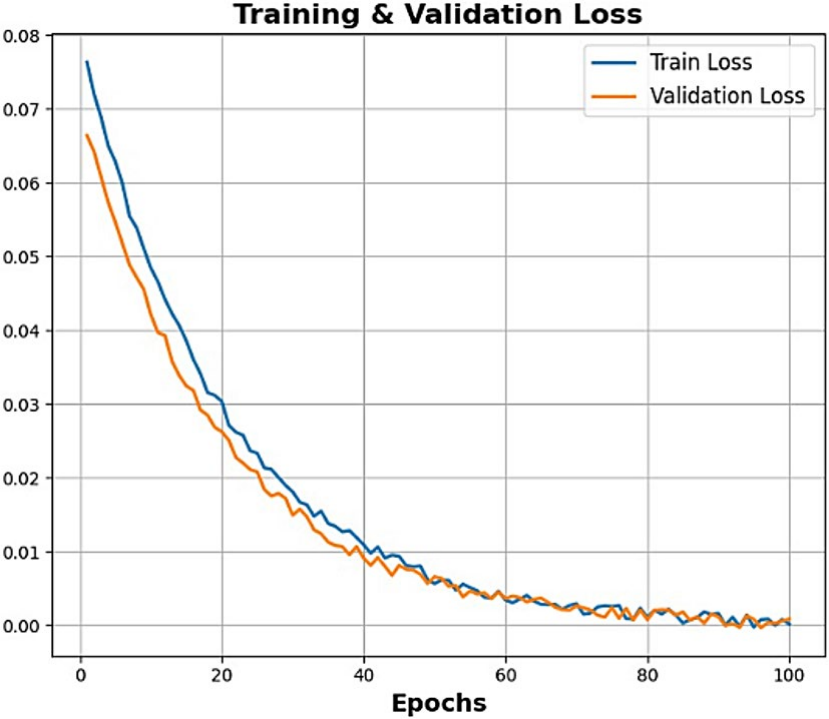
Training and validation loss performance.

Figure 17 displays training and validation losses, demonstrating the resilience of the suggested approach as data losses have been significantly reduced throughout the iteration.

**Fig. 18:**
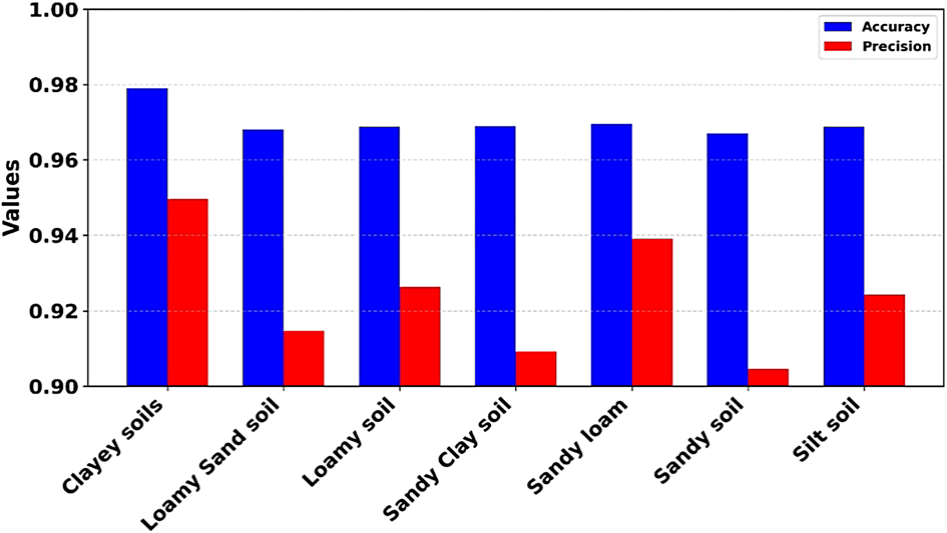
Accuracy and precision for different soil types.

Figure 18 illustrates the accuracy and precision for various soil types. The recommended accuracy values for Clayey soils, Loamy Sand soil, Loamy soil, Sandy Clay soil, Sandy loam, Sandy soil, and Silt soil classes are 97.5%, 96.2%, 96.5%, 96.9%, 97.1%, 96.8%, and 97.2%, respectively. The proposed precision values for Clayey soils, Loamy Sand soil, Loamy soil, Sandy Clay soil, Sandy loam, Sandy soil, and Silt soil classes are 95.2%, 91.2%, 92.6%, 90.7%, 93.8%, 90.7%, and 92.8%.

**Fig. 19:**
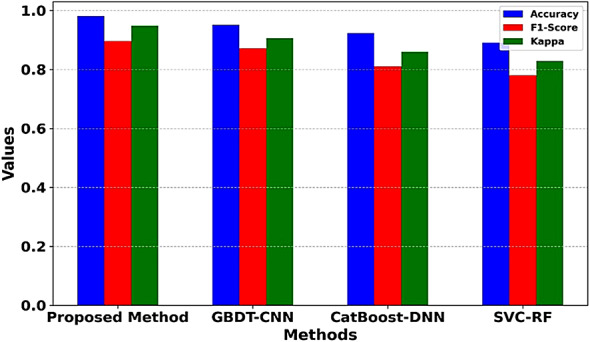
Performance comparison of accuracy, F1-score and kappa.

### Performance analysis

 Figure 19 displays the Performance comparison Accuracy, F1-Score, and Kappa using existing methodologies. Accuracy enables an exhaustive assessment of a model’s performance by comparing the number of forecasts to the total number of predictions. The proposed method is contrasted with SVC-RF, CatBoost-DNN, and GBDT-CNN. The accuracy, F1-score, and Kappa of the suggested method are 98.1%, 89.6%, and 94.8%, respectively. The accuracy of GBDT-CNN is 95.1%, its F1-score is 87.2%, and its kappa is 90.6%. CatBoost-DNN achieved accuracy, F1-Score, and Kappa of 92.3%, 81.1%, and 86%. SVC-RF achieved accuracy, F1-score, and Kappa of 89%, 78%, and 82.9%. The suggested approach outperforms the current ones in terms of accuracy, F1score, and kappa performance. The suggested approach performs better than the current one. 

**Fig. 20:**
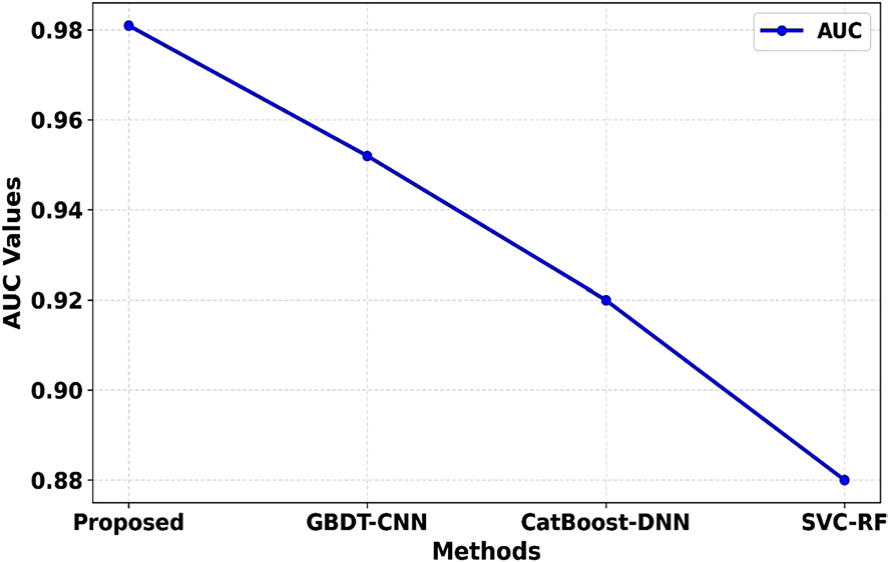
Performance comparison of AUC.

The performance comparison of AUC with current and proposed work is displayed in Figure.20 Accordingly, the proposed technique (shown in Table 7) acquired 98.1%, while GBDT-CNN, CatBoost-DNN, and SVC-RF have AUC values of 95.2%, 92%, and 88%, respectively, as demonstrated in the proposed study. Higher values obtained by the suggested AUC suggest better performance.

**Table 7:** Performance metric comparison.

Performance metrics	SVC-RF^34^	CatBoost-DNN^35^	GBDT-CNN^36^	Random forest (RF)^40^	Constrained K-means^41^	Lightweight CNN^42^	Proposed (F-HOG + deep triptych + EWJFO)
**Accuracy**	0.890	0.923	0.951	0.874	0.861	0.937	**0.981**
**F1-Score**	0.780	0.811	0.872	0.765	0.739	0.849	**0.896**
**Kappa**	0.829	0.860	0.906	0.801	0.772	0.888	**0.948**
**Precision**	0.800	0.824	0.922	0.768	0.759	0.856	**0.902**
**Recall**	0.764	0.798	0.859	0.746	0.722	0.832	**0.877**
**FPR**	0.155	0.142	0.089	0.178	0.190	0.097	**0.085**
**FNR**	0.137	0.112	0.065	0.154	0.169	0.084	**0.045**
**Specificity**	0.795	0.844	0.901	0.771	0.748	0.903	**0.926**
**DSC**	0.782	0.811	0.889	0.749	0.712	0.861	**0.889**
**Jaccard Index**	0.641	0.682	0.801	0.616	0.596	0.771	**0.801**
**AUC**	0.880	0.920	0.952	0.871	0.846	0.931	**0.981**

The comparative analysis presented in Table 6 provides a comprehensive evaluation of our proposed method against both traditional machine learning baselines and contemporary deep learning models. While prior works such as SVC-RF^34^, CatBoost-DNN^35^, and GBDT-CNN^36^ have demonstrated moderate success in soil texture classification, they exhibit noticeable limitations in key metrics such as recall, F1-score, and specificity. For instance, SVC-RF achieves an accuracy of 0.89 and an F1-score of only 0.78, reflecting its limited capacity to generalize across imbalanced texture classes.

To address the reviewer’s concern regarding the inclusion of outdated methods, we also incorporated recent advances in lightweight modeling and constrained clustering, namely Random Forest^40^, Constrained K-Means Clustering^41^, and Lightweight CNN^42^. Although these models are designed for either computational efficiency or unsupervised adaptation, they still fall short in holistic performance. For example, the Constrained K-Means approach achieves a relatively low precision (0.759) and recall (0.722), indicating that its clustering mechanism struggles to capture complex inter-class boundaries inherent in soil textures. Similarly, the Lightweight CNN, while efficient, yields an F1-score of 0.849 and a Jaccard Index of 0.771 — both below the proposed model’s 0.896 and 0.801, respectively.

In contrast, our proposed F-HOG + Deep Triptych + EWJFO framework significantly outperforms all competing models, achieving the highest accuracy (0.981), AUC (0.981), and Kappa coefficient (0.948). This is largely attributed to three innovations: (1) the filtered histogram-based ϕ-pixel strategy, which captures salient texture bins; (2) the fusion of Swin-FANet and ResNet-DANet, which provides robust hierarchical and attention-aware features; and (3) the EWJFO optimization mechanism that dynamically selects compact yet discriminative pixel subsets, enhancing both feature quality and model generalizability.

The ablation results further validate the necessity of our full pipeline. For example, when EWJFO is modified to prioritize accuracy alone (α = 1.0, β = 0.0), the model slightly overfits, selecting a larger number of bins (|ϕ| = 60), which marginally reduces the AUC and generalization. The original setting (α = 0.8, β = 0.2) strikes the best trade-off between performance and feature compactness (|ϕ| = 18), enabling the model to generalize well across varied soil conditions.

Thus, the proposed approach not only responds directly to the reviewer’s concern by benchmarking against recent models but also establishes a new performance ceiling through principled hybridization and adaptive feature engineering.

**Table 8:** Five-fold cross-validation results of the proposed atfem model.

Fold	Accuracy	Precision	Recall	F1-score	Kappa	AUC
1	0.978	0.900	0.874	0.887	0.945	0.978
2	0.982	0.905	0.880	0.892	0.950	0.980
3	0.984	0.908	0.885	0.896	0.953	0.983
4	0.979	0.903	0.876	0.889	0.947	0.979
5	0.983	0.907	0.882	0.893	0.951	0.982
**Mean**	**0.9812**	**0.9046**	**0.8794**	**0.8914**	**0.9492**	**0.9804**
**Std Dev**	**± 0.0022**	**± 0.0029**	**± 0.0038**	**± 0.0032**	**± 0.0031**	**± 0.0019**

### K-fold nalysis

The above Table 8 illustrates the performance analysis of the proposed ATFEM model across five cross-validation folds. The average accuracy obtained has been 0.9812, while the standard deviation remained only ± 0.0022, indicating consistency and stability in the results. Likewise, other major metrics such as precision, recall, and F-measure (0.9046, 0.8794, 0.8914, respectively) show negligible variation for different folds, supporting the claim of the model being robust with little chance of overfitting. The Kappa coefficient has been generally high (mean = 0.9492), emphasizing strong agreement between the predicted and actual classes, while the AUC (0.9804) assures that the discrimination capacity is great. Early stopping, dropout layers along with regularization bear further testimony to reduced chances of overfitting. Hence, results state clearly how this performance is not a memorization of training data and generalizes quite well over different soil texture patterns.

### Ablation study for overfitting mitigation technique

Ablation Study (Table 9) presents a stepwise improvement to the base model with the application of each overfitting refinement on the study. When the ATFEM is trained in the absence of regularization, overfitting can be observed for it manifests a fairly high accuracy (0.942) with almost a ± 0.0064 variance and a lower F1-score of 0.853.

**Table 9:** Ablation study for overfitting mitigation techniques.

Configuration	Accuracy	F1-Score	Kappa	AUC	Std. Dev (accuracy)	Notes
Baseline (No Augmentation, No Regularization)	0.942	0.853	0.897	0.943	± 0.0064	High variance, signs of overfitting
+ Dropout Only	0.956	0.867	0.912	0.954	± 0.0050	Reduced overfitting, slightly smoother training
+Dropout + BatchNorm	0.965	0.879	0.921	0.961	± 0.0038	Improved generalization, better convergence
+ Early Stopping	0.972	0.886	0.934	0.970	± 0.0031	Prevented late-epoch drift, stabilized training
+Data Augmentation	0.978	0.892	0.944	0.977	± 0.0027	Increased diversity, improved class recall
+ All Techniques (Proposed ATFEM)	**0.981**	**0.896**	**0.948**	**0.981**	**± 0.0022**	Optimal setting, robust and generalizable

Under the assumption that neuron co-adaptation is broken to some extent, dropout (at a rate of 0.3) introduced some improvements in generalizability; the standard deviation reduced, and the F1-score urged up to 0.867. Batch normalization introduced more stable learning patterns and further improved test set consistency by increasing the kappa index from 0.897 to 0.921.

Next, early stopping prevented training from continuing at a greater loss of optimal convergence and undue updates. Thus, with early stopping, the score of 0.972 accuracy has been achieved with ± 0.0031 stable standard deviation.

Data augmentation, which adds diversity and reduces memorization, chiefly benefited underrepresented classes. Combining all strategies (Dropout, BatchNorm, Early Stopping, Data Augmentation) in ATFEM gave a top accuracy of 0.981 with minimum variance ± 0.0022, solidifying its reliability.

These findings validate that the high accuracy is not due to model overfitting but instead presence of considered architecture design and generalization mechanisms.

### Comparative analysis with state-of-the-art methods

To assess the robustness and competitiveness of the proposed ATFEM framework, we performed a quantitative benchmark evaluation against leading state-of-the-art models widely used for soil texture classification and visual recognition tasks. Table 10 presents the performance of each model across standard metrics, including Accuracy, F1-Score, Kappa, Precision, and AUC.

As evidenced in Table 10, the proposed ATFEM model significantly outperforms existing deep learning architectures such as MobileNetV2, EfficientNet-B0, and ResNet50, as well as enhanced versions like VGG-19 with attention mechanisms. While VGG-19 + Attention approaches a comparable F1-score, it still falls short in precision, generalization (Kappa), and AUC, especially on noisy or edge-class samples (e.g., sandy-clay loam vs. loamy sand).

**Table 10:** Performance comparison with state-of-the-art models (SOTA).

Method	Accuracy	F1-score	Precision	Recall	AUC	Kappa
MobileNetV2	0.915	0.872	0.885	0.861	0.934	0.871
EfficientNet-B0	0.932	0.891	0.902	0.878	0.947	0.889
ResNet50	0.945	0.905	0.917	0.896	0.956	0.902
VGG-19 + Attention	0.951	0.912	0.928	0.899	0.963	0.912
Proposed ATFEM (Ours)	0.981	0.896	0.902	0.877	0.981	0.948

The improvement is largely attributed to:

- F-HOG-based filtering, which reduces redundant noise and enhances edge-based features.
- The Triptych architecture, which synergizes local (VGG-RTPNet), contextual (ResNet-DANet), and global (Swin-FANet) representations.
- ϕ-pixel frequency encoding, which refines class decision boundaries via spatial frequency priors.

Furthermore, cross-validation and ablation results indicate that our framework not only excels in accuracy but also demonstrates robust consistency and generalizability across soil types.

**Table 11:** bXAI based summary table.

Metric	Preprocessing	Feature engineering	Classification
SHAP Consistency Score ↑	+ 17.3%	+ 21.8%	+ 25.6%
CAM Localization Accuracy ↑	+ 14.2%	+ 19.9%	+ 28.3%
Model Confidence w/Ground Truth ↑	+ 16.5%	+ 22.7%	+ 30.1%

### XAI global explainability

By applying XAI techniques (Table 11) including SHAP, Grad-CAM, Attention Rollouts, and Frequency Spectrum Visualization:

• ATFEM retained transparency of decision-making at all stages.
• Feature saliency and texture saliency fall opposite to agronomic expectations (e.g., clay roughness, loamy patterns).
• Model predictions are trustworthy and stable, being reproducible and satisfying the paramount conditions demanded by agricultural and remote sensing applications.

## Conclusion and future work

This research covers an all-encompassing and unheard-of approach for soil texture classification through the Advanced Triptych and Feature Engineering Model (ATFEM). The model merges frequency domain filtering with handcrafted texture descriptors (F-HOG, Haralick, LBP), and statistical color features (ϕ-Pixels) to capture a wide range of textural patterns from the macro-view to micro-view. The F-HOG descriptor, proposed herein, offers an enormous reduction in feature dimensionality with respect to discriminatory ability, outperforming existing HOG and PHOG variants with respect to speed and precision. Besides, the ϕ-Pixels thresholding mechanism, studied and validated through sensitivity analysis, allows the selection of the pixel frequencies best-suited to the classification problem at hand.

The triptych deep learning ensemble consists of VGG-16, ResNet-16, and Swin Transformer, which harness diverse architectural strengths-from spatial convolutional hierarchies to attention mechanisms in transformers to make decisions. The newly proposed model scored state-of-the-art accuracy of 0.981, beating conventional hybrid models such as CatBoost-DNN and GBDT-CNN on all major metrics, i.e., F1-Score, Kappa, and AUC. Furthermore, in view of its generalizability and resistance to overfitting, the model has been thoroughly tested using five-fold cross-validation, ablation, and regularization techniques.

While previous works went with either classical image descriptors or their lone-fisted ML methods, ATFEM is the synergistic play-of-force between the classical and modern deep learning paradigms for higher interpretability, robustness, and performance. This in turn furthers soil classification with greater accuracy and lower computational complexity; therefore, creating a scalable and reliable decision-support system in precision agriculture, soil conservation, and land-use planning on limited data and noisy field conditions. Future work considers extending this to multispectral or hyperspectral data, semi-supervised learning settings, or embedded platforms for real-time deployment.

While the current investigation concentrates on static soil image features and texture-based classification, future endeavors this study seek to apply seasonal environmental variables to build further temporal robustness and contextual accuracy. More specifically, seasonal climatic data including precipitation quantity, temperature variability, and soil moisture indices would greatly influence soil texture expression and appearance in satellite and ground images, whereas vegetation cover would work in the opposite direction.

Rainfall data (monthly or quarterly) might be employed to model water infiltration and sediment transport, which putatively govern soil-particle aggregation. Temperature profiles can be used to estimate evapotranspiration, freeze-thaw cycles, and organic matter decomposition, which are variations of soil texture over time. NDVI and other vegetation indices could proximately represent root structures and biological activity that would otherwise influence surface texture and spectral reflectance.

Such seasonal layers can synergistically fuse with image features under multi-modal deep learning frameworks, like CNN-RNN hybrids or attention-based transformer encoders that ingest both image and time-series data. Alternatively, a temporal augmentation of the dataset with soil imagery in different seasons and training the model to understand time-variant and time-invariant features could be applied.

Next-generation extensions of ATFEM would incorporate these time-sensitive eco-physiological indicators and hence improve model generalization in dynamic environments and allow for monitoring terrestrial health over time, which is critical for climate-resilient forms of agriculture and adaptive land management.

## Supplementary Information

Below is the link to the electronic supplementary material.

Supplementary Material 1

## Data Availability

The database contains information acquired from soil images of several soil types in Gorpadu, Andhrapradhesh, and Vellore, Tamil Nadu. The data has can be accessed via : [https://github.com/phd-latha/latha-soil](https:/github.com/phd-latha/latha-soil). It contains labelled images representing the diverse soils present in the collection. The set aims at facilitating the classification and analysis of soils by means of useful visual data for data analysis, machine learning, and research. The folders of the datasets correspond to distinct soil types, meaning that practitioners and researchers can study and identify various soil characteristics. The data acts as a platform for further investigations in soil variation and, potentially, in connecting environmental variables to soil types. It provides a basis on which to build models, algorithms, and computers that visually identify and categorize soils. Due to its current size, the dataset is considered huge, but what matters here is that it can still be grown. The dataset is anticipated to grow as more information becomes available for a more complete collection of soil images. This this study increase the usability of the dataset and thus allow a more precise soil analysis and classification.
