# Supplementary material for: Advanced deep learning framework for soil texture classification
Source: Sci Rep. 2025 Oct 2;15:34407. doi: 10.1038/s41598-025-17384-5 (PMC12491579; doi:10.1038/s41598-025-17384-5)
Supplement: Supplementary file 1 — Supplementary Material 1 [file 41598_2025_17384_MOESM1_ESM.docx]

**Appendix**


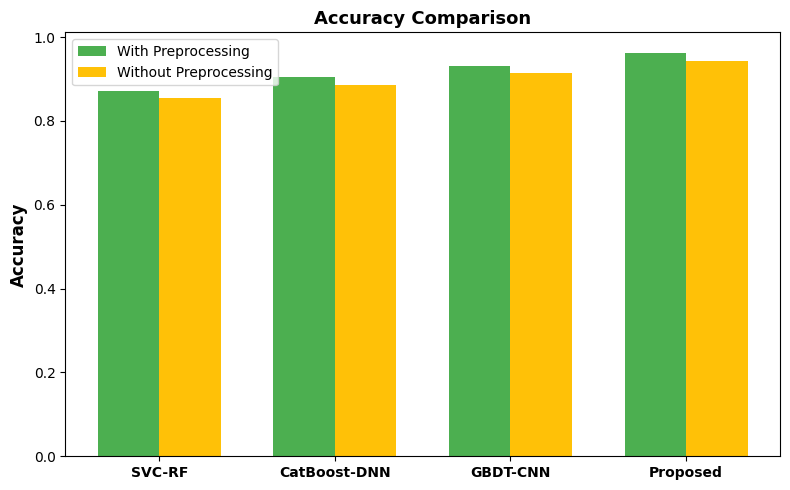


1. **Accuracy comparison**


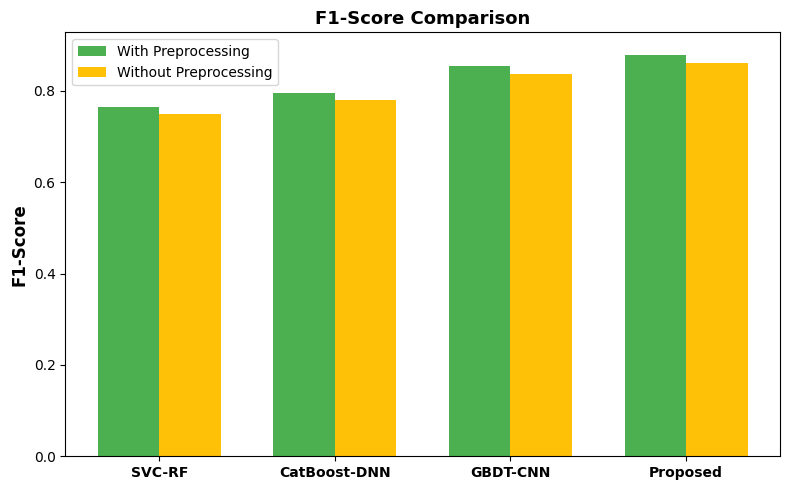


1. **F1-Score comparison**


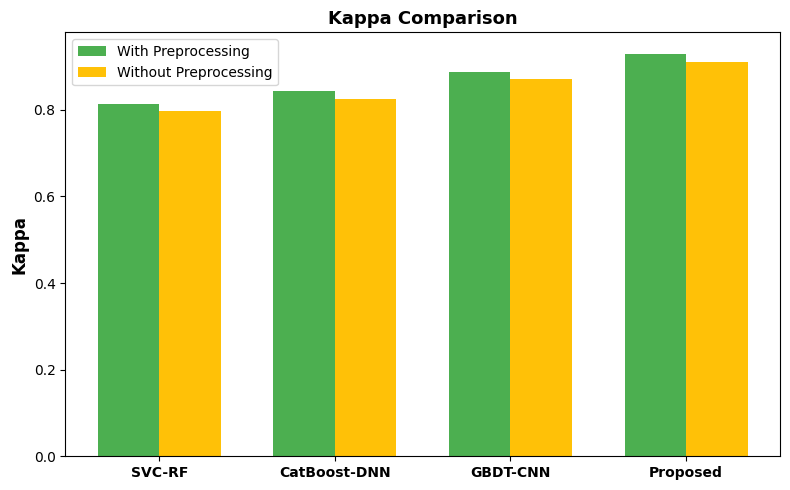


1. **Kappa Comparison**


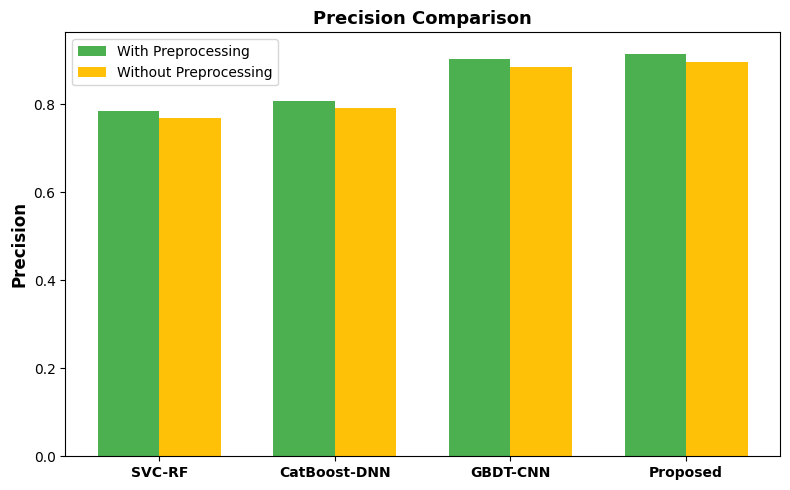


1. **Precision Comparison**


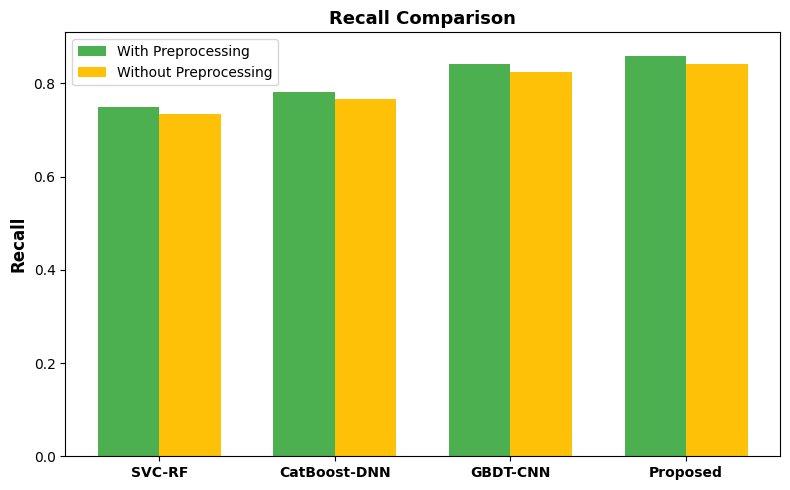


1. **Recall Comparison**


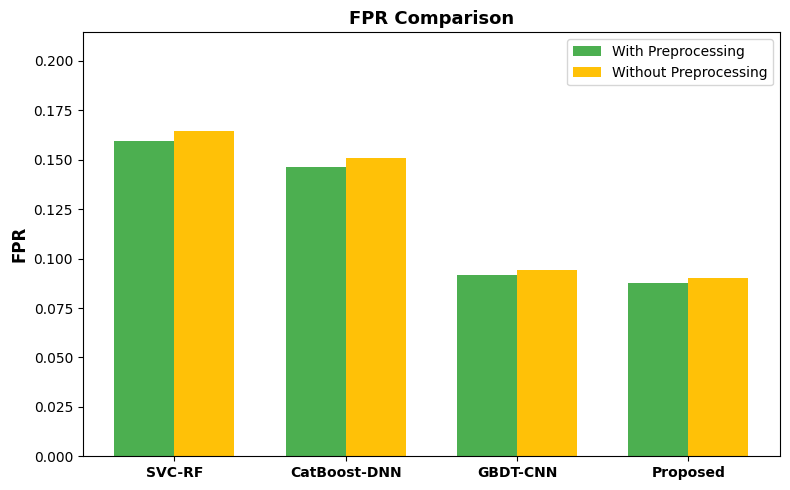


1. **FPR Comparison**


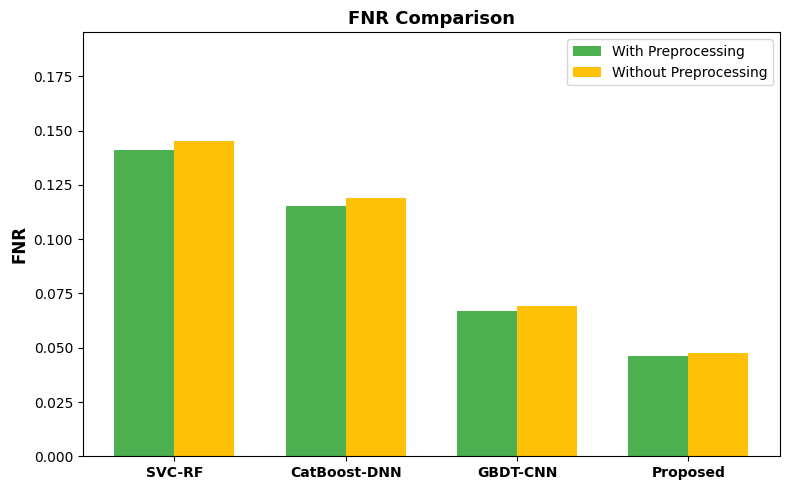


1. **FNR Comparison**


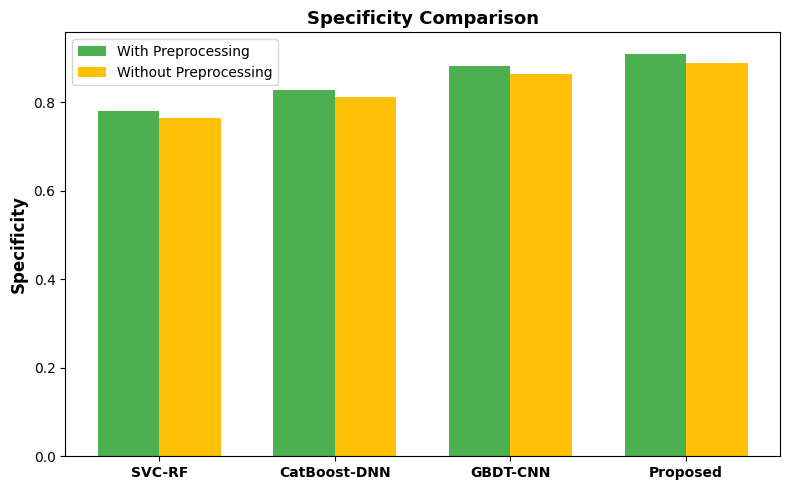


1. **Specific Comparison**


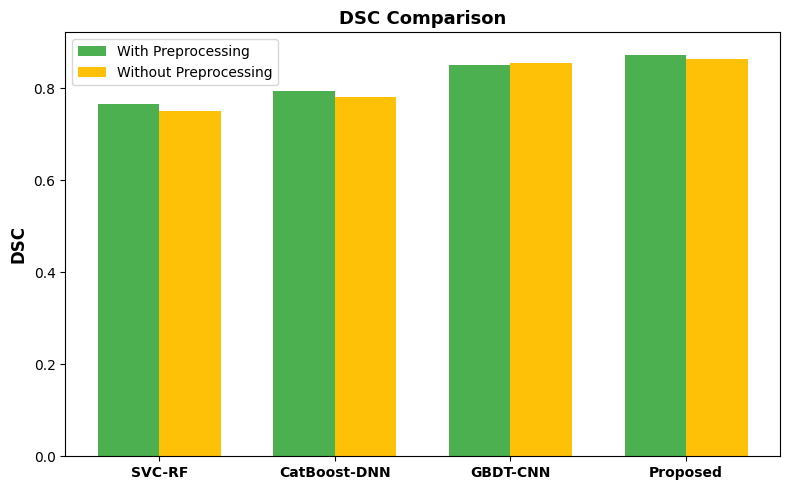


1. **DSC Comparison**


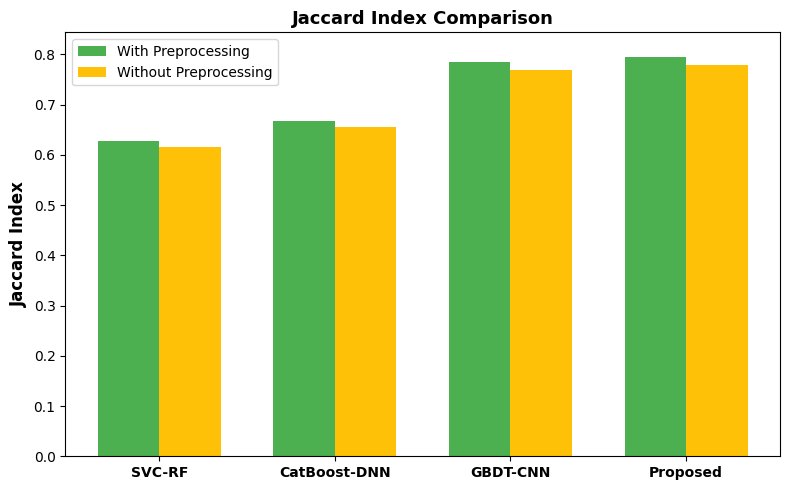


1. **Jaccard Index Comparison**


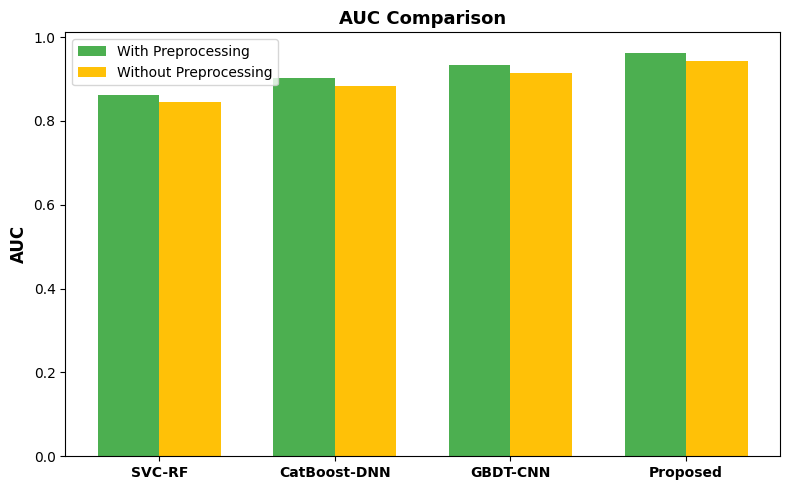


1. **AUC comparison**

**Supplementary Figure S1: Performance analysis – with/ without pre-processing**


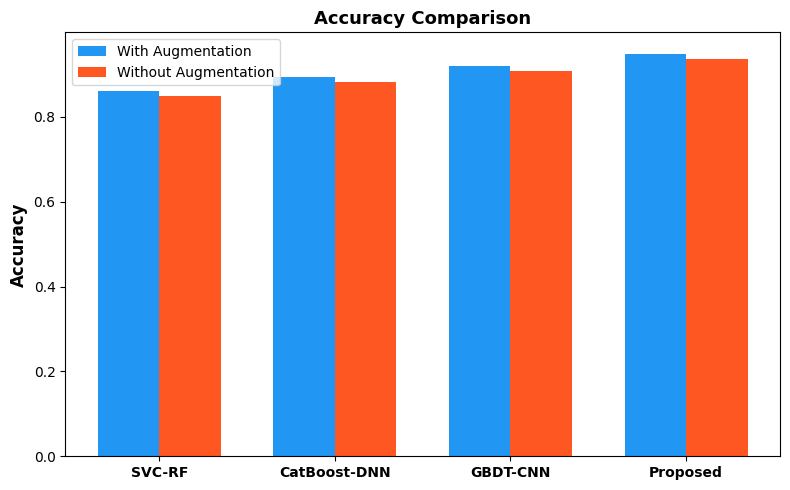


1. **Accuracy Comparison**


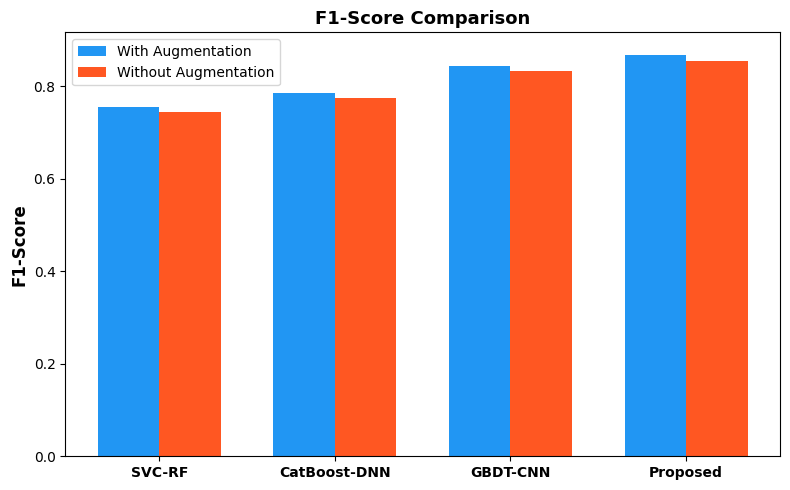

**(b) F1-Score Comparison**


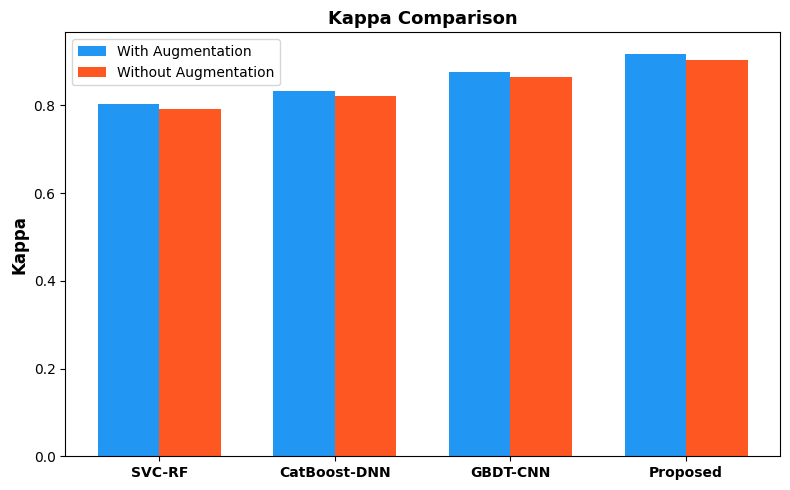


1. **Kapp Comparison**


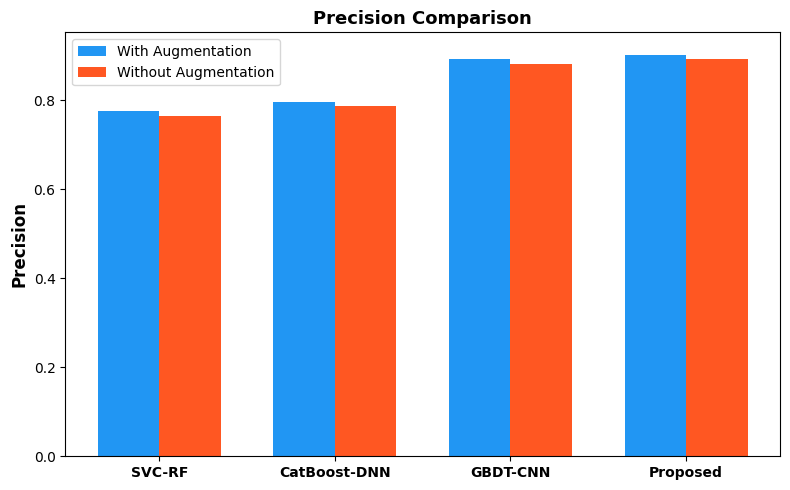


1. **Precision Comparison**


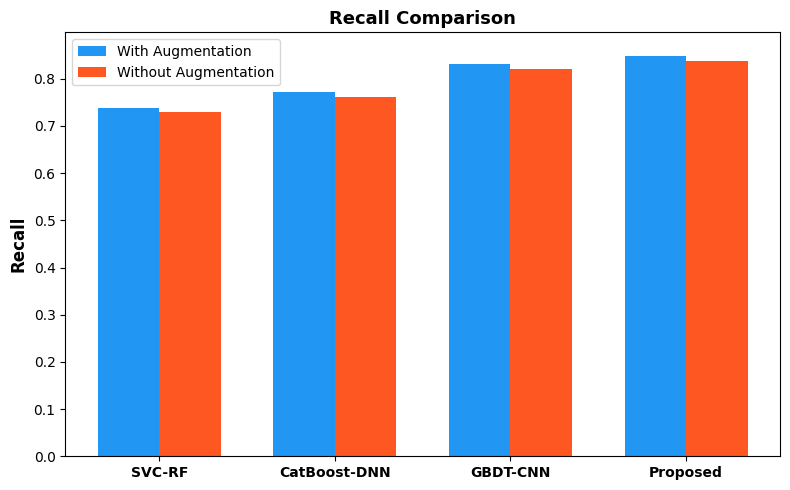


1. **Recall Comparison**


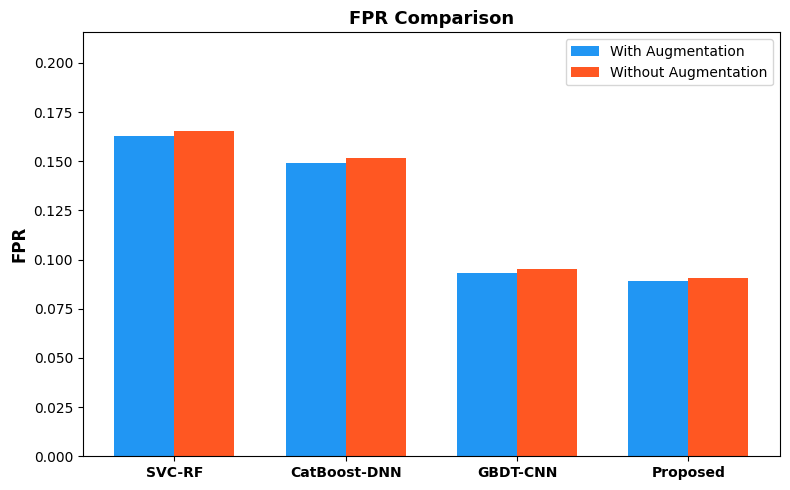


1. **FPR Comparison**


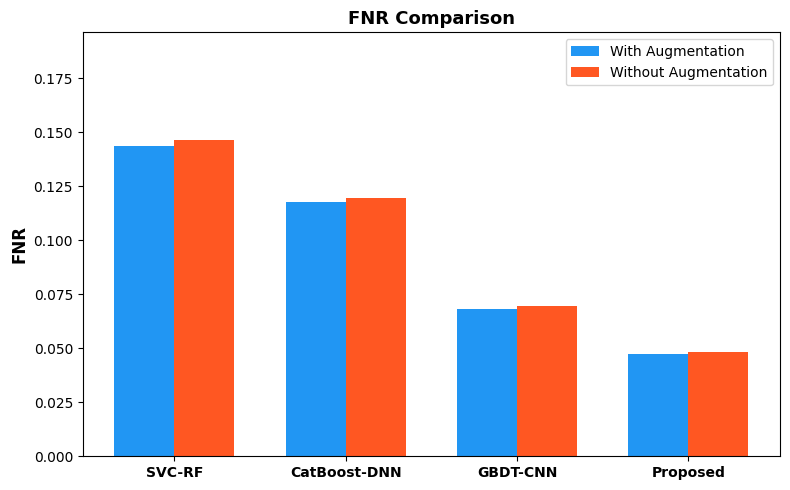


1. **FNR Comparison**


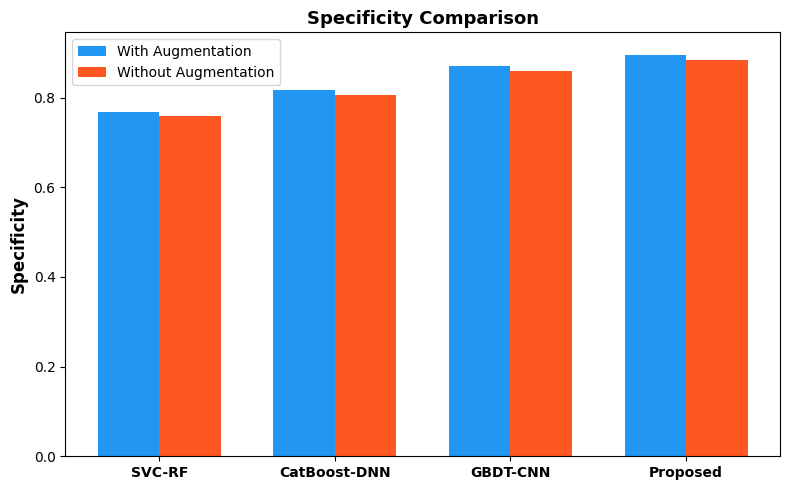


1. **Specificity Comparison**


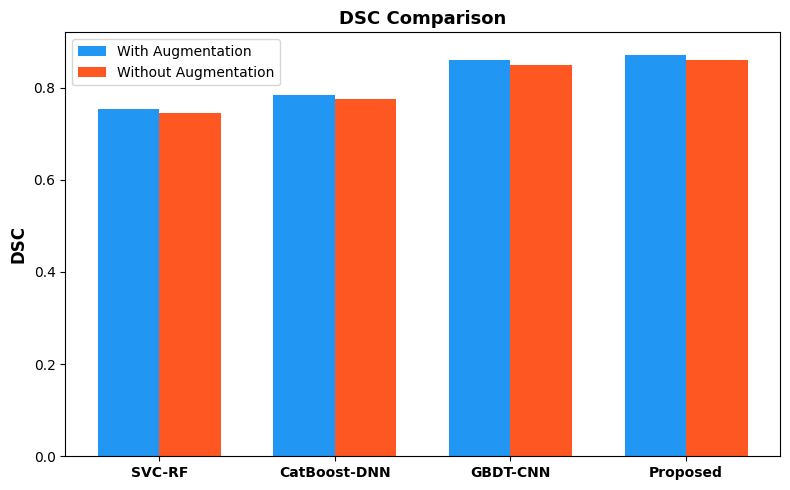


1. **DSC Comparison**


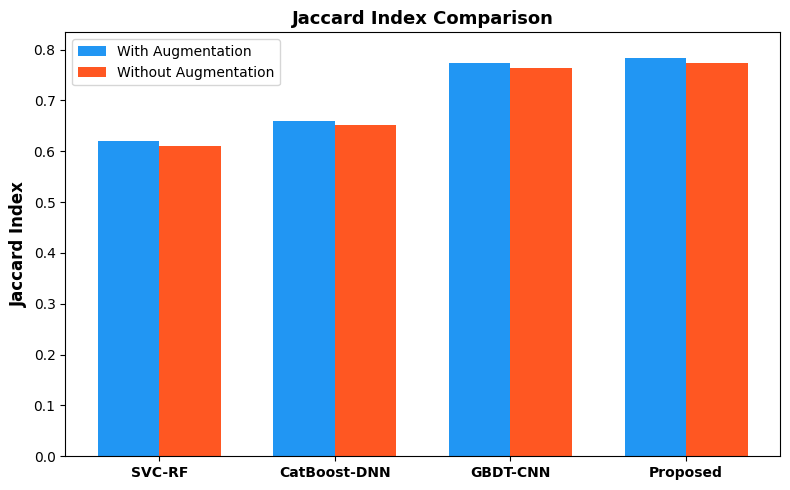


1. **Jaccard Index Comparison**


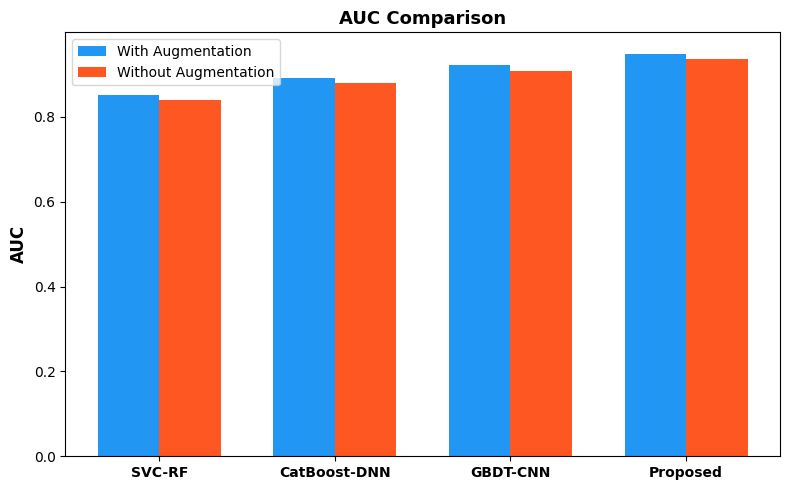


1. **ACU Comparison**

**Supplementary Figure S2: Augmentation stage- with/ without**


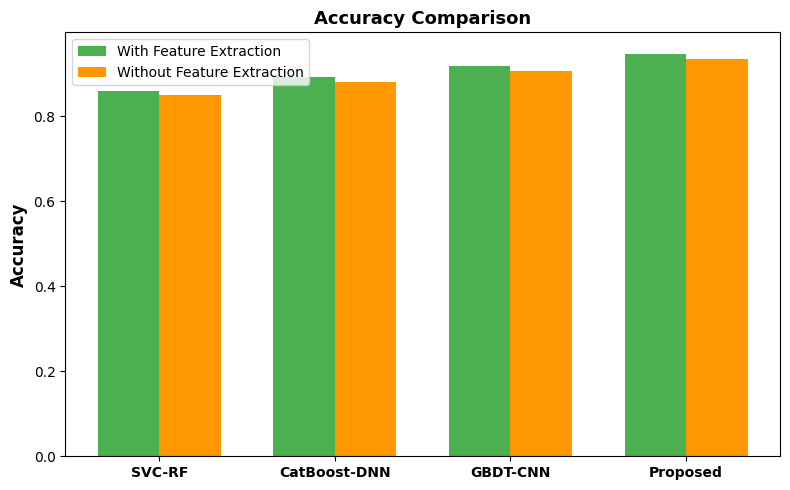


1. **Accuracy Comparison (Feature Extraction)**


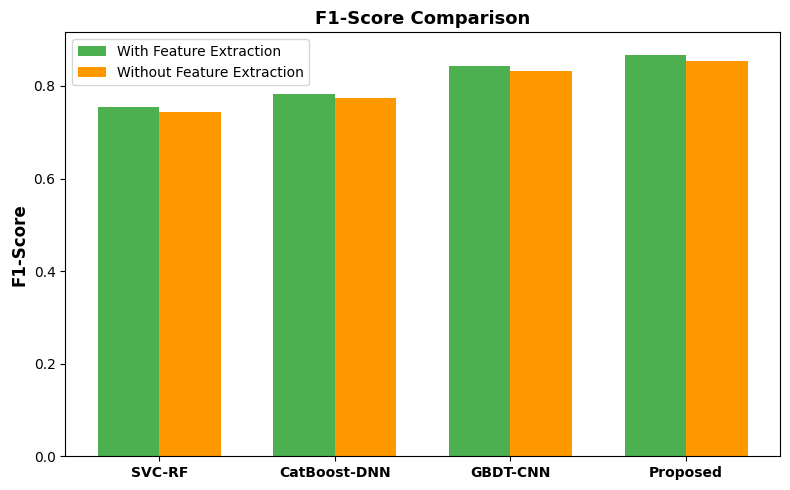


1. **F1-Score Comparison (Feature Extraction)**


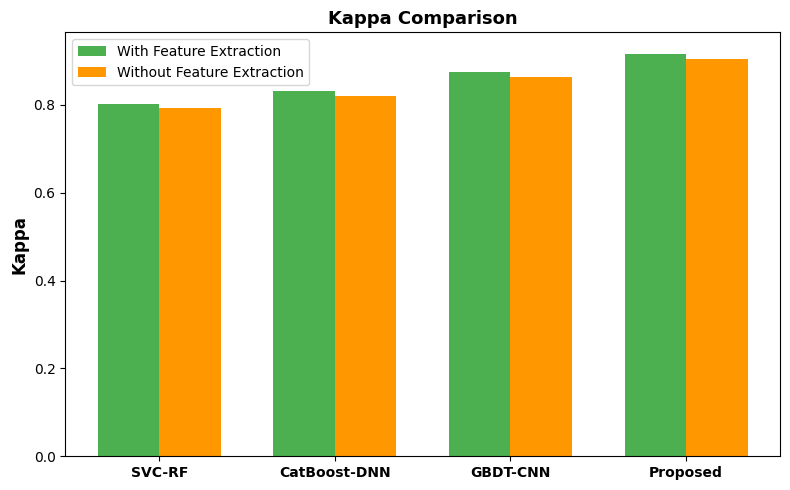


1. **Kappa Comparison (Feature Extraction)**


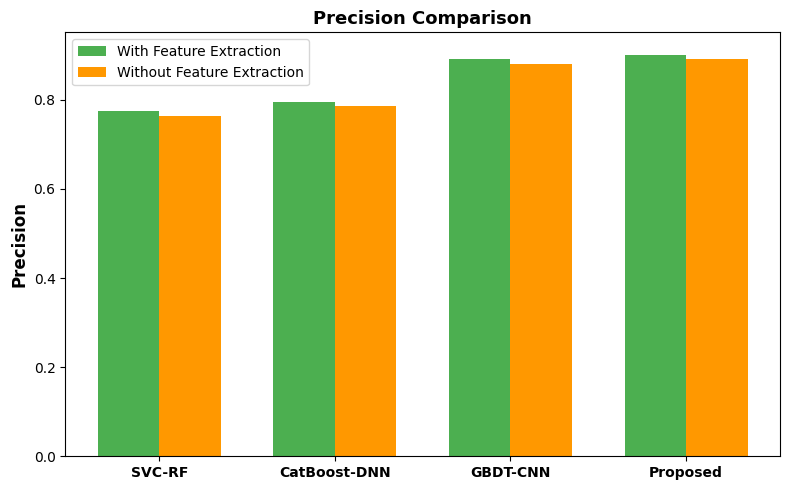


1. **Precision Comparison (Feature Extraction)**


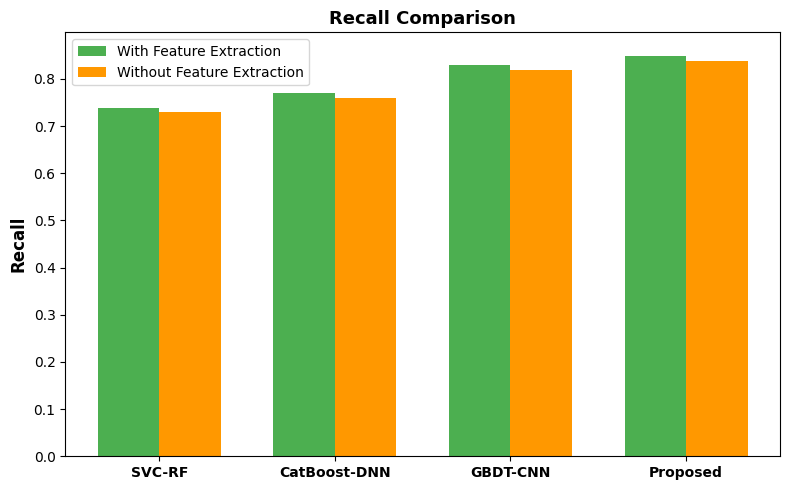


1. **Recall Comparison (Feature Extraction)**


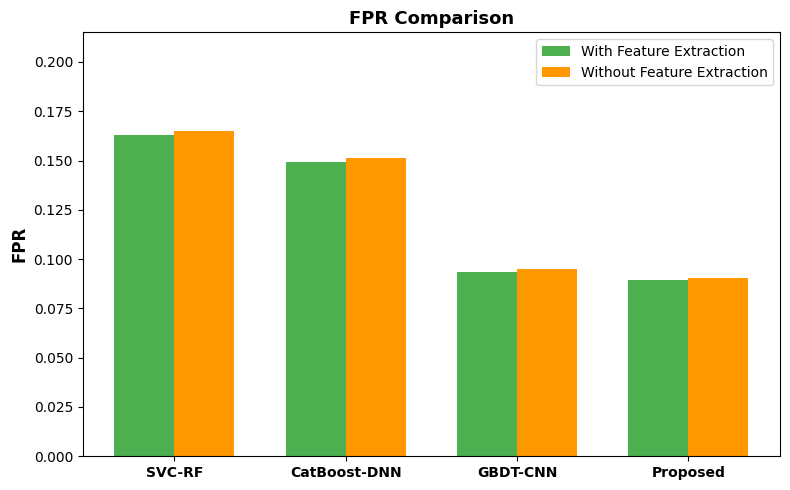


1. **FPR Comparison (Feature Extraction)**


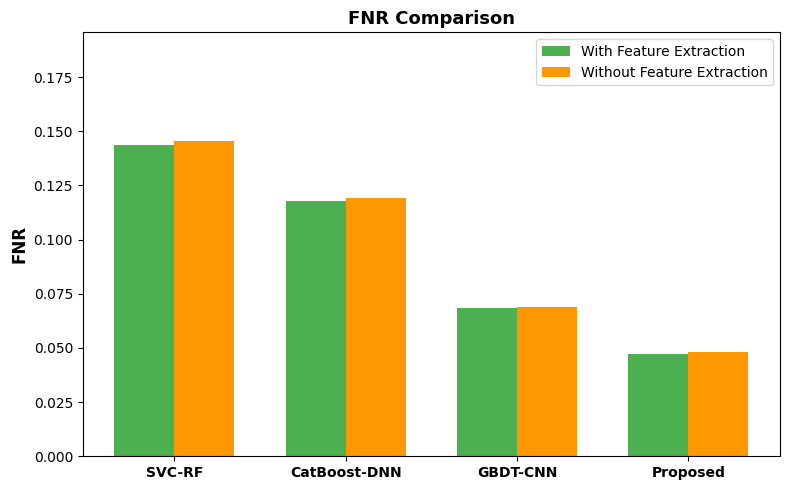


1. **FNR Comparison (Feature Extraction)**


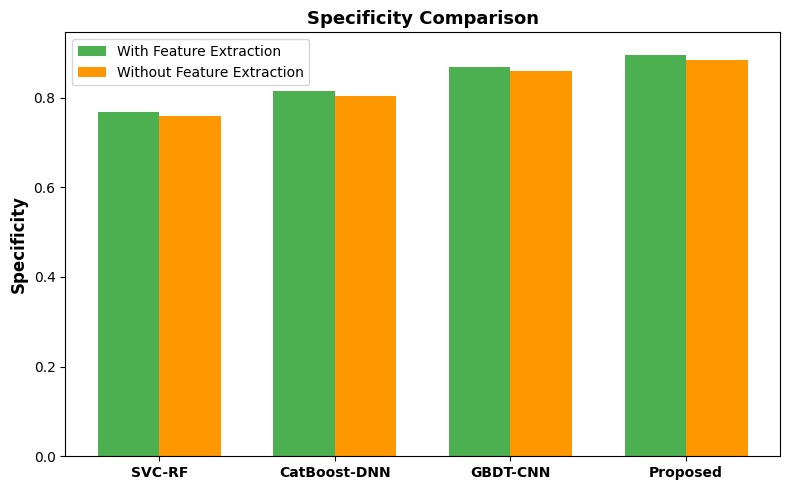


1. **Specific Comparison (Feature Extraction)**


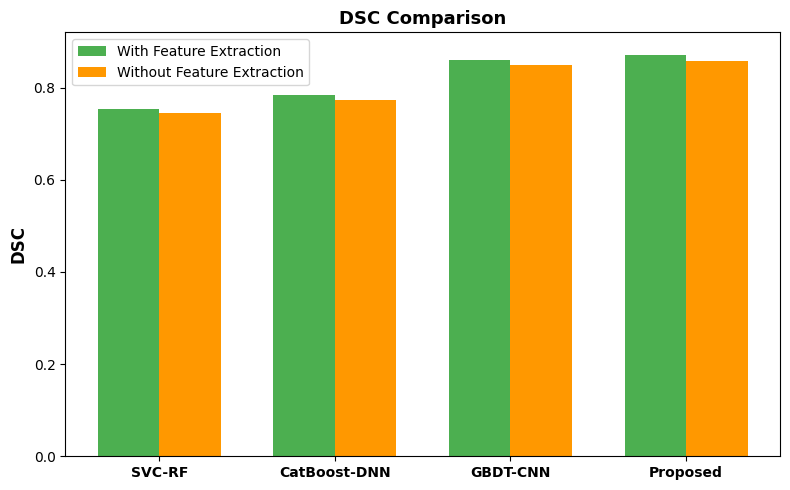


1. **DSC Comparison (Feature Extraction)**


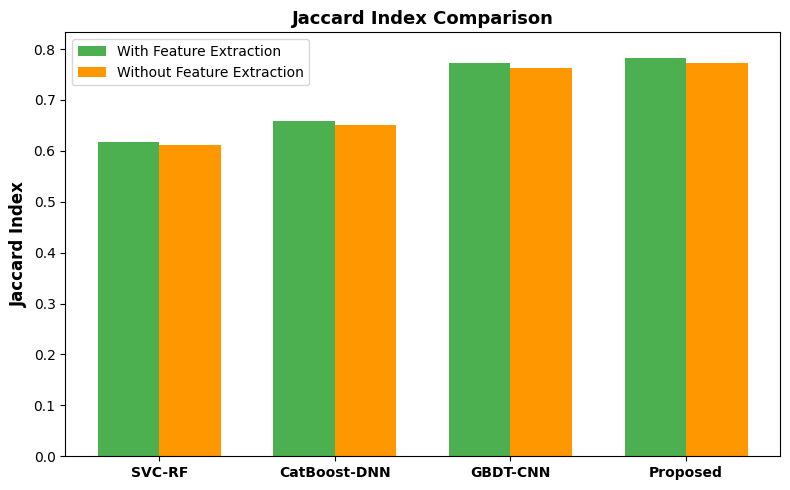


1. **Jaccard Index Comparison (Feature Extraction)**


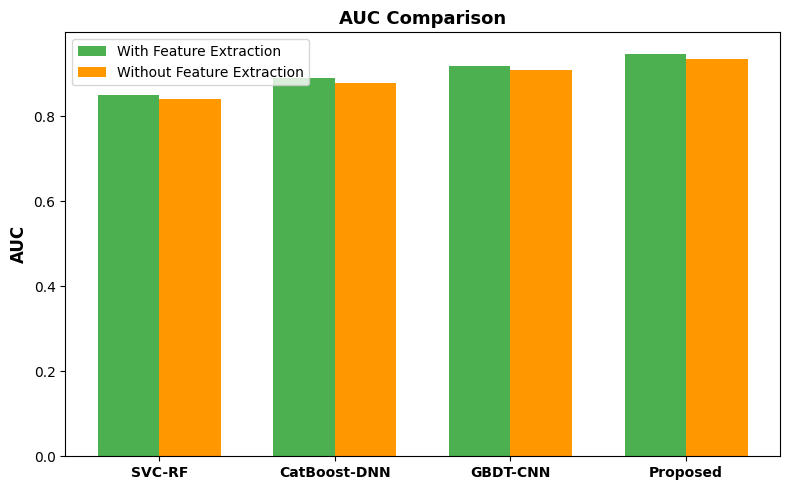


1. **AUC Comparison (Feature Extraction)**

**Supplementary Figure S3:**  Analysis on feature extraction stage – with/ without
